# Fine mapping and candidate gene analysis of *qKnps-2A* for kernel number per spike in wheat

**DOI:** 10.1186/s12870-026-09727-9

**Published:** 2026-08-11

**Authors:** Meijie Zhu, Yongzhen Wu, Guimei Hu, Dengke Xu, Tong Li, Yifan Wang, Chenxiao Cui, Yu Wang, Jia Zhou, Chunhua Zhao, Ran Qin, Zuodong Wang, Daoquan Cheng, Jianjian Zhang, Han Sun, Junming Li, Fa Cui

**Affiliations:** 1 College of Horticulture, Yantai Key Laboratory of Molecular Breeding for High-Yield and Stress-Resistant Crops and Efficient Cultivation, Ludong University, Yantai, 264025 China; 2 Ministry of Education Key Laboratory of Molecular and Cellular Biology, Hebei Collaboration Innovation Center for Cell Signaling and Environmental Adaptation, Hebei Key Laboratory of Molecular and Cellular Biology, College of Life Sciences, Hebei Normal University, Shijiazhuang, 050000 China; 3 Binzhou Key Laboratory of Crop Stress Resistance & Immune Regulation, Shandong Jingbo Agrochemicals Technology Co., Ltd., Binzhou, 256500 China

**Keywords:** Wheat, Kernel number per spike, Fine mapping, Candidate gene analysis, Haplotype analysis

## Abstract

**Background:**

Kernel number per spike (KNPS) is a crucial determinant of wheat yield. Previous studies identified *qKnps-2A* as a major stable QTL for KNPS on chromosome 2A using a recombinant inbred line (RIL) population derived from a cross between the semi-winter wheat cultivar Kenong 9204 and the winter wheat cultivar Jing 411. However, fine mapping and gene cloning of this locus have not been reported.

**Results:**

Using InDel markers developed from parental genomic data, the near-isogenic line (NIL) pairs and 11 key recombinants were identified from residual heterozygous line-derived segregating populations, delimiting *qKnps-2A* to a 5.13 Mb interval (616.30–621.43 Mb) harboring 50 high-confidence genes. Integrated transcriptome sequencing, expression profiling across tissues and developmental stages, sequence comparison, and haplotype analysis identified *TraesCS2A03G0902100*, encoding a zinc knuckle protein, as the most likely candidate gene. Near-isogenic line analysis revealed that the Kenong 9204 allele significantly increased spikelet number per spike (2.94%), floret number per spike (10.51%), and KNPS (14.37%), resulting in a 7.32% yield increase, alongside improvements in grain quality traits. Phytohormone analysis showed the Kenong 9204 allele was associated with elevated IAA and IAA/ABA ratios during early spike development and reduced ABA during late development, suggesting hormone-related physiological changes linked to improved grain set. Haplotype analysis of 314 global accessions showed that Hap-KN9204 was significantly associated with increased KNPS, grain compactness, grain setting, and grain width. This superior haplotype predominates in Asia, Europe, and North America, and is suggestive of positive selection during Chinese wheat breeding, increasing from 30.00% in landraces to 59.62% in modern cultivars.

**Conclusion:**

This study fine-mapped *qKnps-2A* and identified *TraesCS2A03G0902100* as the most likely candidate gene, which was associated with altered phytohormone profiles during spike development, suggesting physiological changes linked to enhanced yield. The superior Hap-KN9204 haplotype appears to have been favored by positive selection in Chinese wheat breeding, establishing *qKnps-2A* as a valuable target for yield improvement.

**Supplementary Information:**

The online version contains supplementary material available at https://doi.org/10.1186/s12870-026-09727-9.

## Introduction

Wheat is a major food crop globally. China currently leads the world in both wheat production and consumption, accounting for approximately 17.8% to global wheat output [1, 2]. Increasing grain yield is the primary objective of wheat breeding. Yield, a complex quantitative trait that is controlled by many loci, is largely determined by three components: spike number per unit area, kernel number per spike (KNPS), and thousand kernel weight (TKW). Among these, KNPS is pivotal, and dissecting its genetic and molecular basis offers a direct route to break the current yield ceiling in wheat.

KNPS is primarily governed by spikelet number per spike (SNPS) and grain number per spikelet [3]. Manipulating spikelet number and floret fertility can indirectly modulate KNPS, thereby exerting a significant impact on wheat yield. Extensive studies have identified numerous genes regulating these processes. For spikelet initiation and development, *FRIZZY PANICLE* (*WFZP*) promotes spikelet initiation and development by activating *VRN1* and *HOMEOBOX4* [4] and represses *TaBA1* while activating awn development genes [5]; a paired spikelet QTL on chromosome 4D [6] was attributed to *TEOSINTE BRANCHED1* (*TB1*) allelic diversity, which affects paired spikelet development and tiller number [7]; a deletion in *FT* is significantly associated with spikelet number and heading date [8]; and *TaCOL-B5* regulates spikelet number, tiller number and grain yield per plant [9]. For floret fertility and spikelet-axis growth, *Grain Number Increase 1* (*GNI1*) encodes a homeodomain-leucine zipper class I (HD-Zip I) transcription factor that suppresses apical floret development and modulates spikelet-axis growth, thereby determining KNPS [10]; *TaGW2-A1* affects KNPS, TKW and grain morphology via reduced promoter activity [11]; and down-regulation of *TaDEP1* likely optimizes spike development by modulating auxin transport and distribution [12]. Reverse-genetic strategies have progressively revealed the genes and regulatory mechanisms underlying KNPS and SNPS in wheat. *TaAGL6* increases SNPS and consequently KNPS when overexpressed [13]; the *dep1-B* mutant exhibits a significant reduction in SNPS [13]; overexpression of *TERMINAL FLOWER 1-2D* (*TaTFL1*) prolongs the double-ridge and floret phases, significantly increasing SNPS, FNPS, and KNPS [14]; *TaCKX2.1* and *TaCKX2.2* transcript abundance is highly significantly and positively correlated with KNPS [15]; and down-regulation of *TaPIN1s* expression alters SNPS and KNPS while increasing grain yield [16]. These advances have defined key molecular regulators of spike development and provided valuable targets for breeding; yet the causal genes and mechanisms within mapped QTL intervals remain to be elucidated.

Complementing these gene-level advances, QTL mapping has been extensively applied to elucidate the genetic basis of KNPS. On chromosome 2 A, several distinct loci have been independently identified. Li et al. [17] identified 46 QTLs associated with yield traits distributed across 12 chromosomes; among them, the KNPS QTL *QKns.sdau-2A* on chromosome 2 A was consistently detected across multiple environments. Cui et al. [18] identified 17 QTLs associated with KNPS, among which *QKnps-2A.1* and *QKnps-2A.2* were located on chromosome 2 A, with the latter being a stable and major-effect QTL. Bhusal et al. [19] identified six QTLs for KNPS under both optimal and heat-stress conditions, explaining 7.20–23.16% of the phenotypic variance. Shi et al. [20] identified 47 loci significantly associated with KNPS; among them, the overlapping locus BS00022896_51 on chromosome 2 A was detected by both association and linkage analyses, and its physical position is close to that identified in our previous study [21]. Lee et al. [22] identified the KNPS QTL *Xgpw2206* on chromosome 2 A, explaining 20.4% of the phenotypic variance. Four QTL-rich clusters (QRCs) associated with KNPS have been identified on wheat chromosome 2 A as hotspot intervals for subsequent gene cloning and marker-assisted breeding [23]. Nevertheless, these loci were predominantly defined by primary mapping with large confidence intervals, leaving their candidate genes and molecular mechanisms unresolved. Notably, our previous study identified a major stable QTL, *qKnps-2A*, on chromosome 2 A using a RIL population derived from Kenong 9204 (KN9204) × Jing 411 (J411), which was consistently detected across six environments, explaining 7.07–14.38% of the phenotypic variance [21]. Comparative physical mapping indicates that the *qKnps-2A* interval does not overlap with any previously identified KNPS-related loci on chromosome 2 A [17–19, 22, 23]. Notably, *qKnps-2A* is physically close to BS00022896_51 [20]; however, fine mapping and gene cloning at this locus remain unreported. Therefore, the causal gene(s) within *qKnps-2A* and their molecular mechanism remain critical unanswered questions.

To address this limitation, the present study conducted fine mapping and candidate gene analysis of *qKnps-2A*. Haplotypes and genetic effects were further evaluated using a diverse natural population. The possible molecular and physiological basis of *qKnps-2A* was explored through near-isogenic line analysis, transcriptome sequencing, and phytohormone analysis. These findings establish *qKnps-2A* as a valuable locus for high-yield wheat breeding and provide theoretical insights into the genetic basis of yield formation.

## Materials and methods

### Residual heterozygote screening and fine mapping of *qKnps-2A*

Residual heterozygous lines (RHLs) used for fine mapping of *qKnps-2A* were screened from a primary mapping population consisting of 427 F₇ recombinant inbred lines derived from a cross between the semi-winter wheat cultivar Kenong 9204 and the winter wheat cultivar Jing 411. The wheat cultivars KN9204 and J411, along with the derived recombinant inbred line population (KJ-RILs), were provided by the Center for Agricultural Resources Research, Institute of Genetics and Developmental Biology, Chinese Academy of Sciences, Shijiazhuang, China. The screening criteria for RHLs required that the target QTL region remained heterozygous while other regions of the genetic background were homozygous. The selected RHLs were self-pollinated to produce F₈ segregating populations for subsequent fine mapping. InDels (≥ 5 bp) between parents were screened based on the KN9204 genome [24] and the re-sequencing data of J411. Polymorphic molecular markers (Table S1) were developed using the PrimerServer tool (http://wheatomics.sdau.edu.cn/PrimerServer/) on the WheatOmics platform to identify plant genotypes [25].

Recombinant individuals from the F₈ generation were genotyped using polymorphic molecular markers targeting the interval and its flanking regions. Combined with KNPS phenotype data, *qKnps-2A* was delimited to a narrower interval. Based on this, near-isogenic lines (NILs) with homozygous target regions and consistent genetic backgrounds were obtained through multiple generations of self-pollination and marker-assisted selection. NIL families homozygous for KN9204 and J411 genotypes at the target region were designated as NIL-KN9204 and NIL-J411, respectively. Transcriptome-based genotyping across over 150,000 high-confidence SNP sites revealed genetic similarities of 98.12% (EDS) and 98.34% (LDS) between NIL-KN9204 and NIL-J411 (Table S2). Sliding-window analysis (1 Mb window, ≥ 5 SNPs threshold) further showed that residual donor polymorphisms were almost exclusively confined to a dense block on Chr2A, with no detectable donor segments on the remaining 20 chromosomes (Fig. S1). These results confirm a highly recovered recurrent background and that the phenotypic variation is driven by the target *qKnps-2A* locus. At maturity, twenty plants were selected from each of the NIL-KN9204 and NIL-J411 near-isogenic lines to investigate agronomic traits: plant height (PH), spike length (SL), spikelet number per spike (SNPS), floret number per spike (FNPS), kernel number per spike (KNPS), thousand kernel weight (TKW), grain length (GL), and grain width (GW). PH was measured from the stem base to the top of the main tiller spike (excluding awns). SL was measured from the base of the spike to the tip (excluding awns). SNPS, FNPS, and KNPS were measured on the main tiller spike. TKW, GL, and GW were determined using the Intelligent Test and Analysis System (TOP Cloud-agri Technology, Zhejiang, China) from seeds after harvest. This system integrates image analysis and precision weighing modules, enabling simultaneous measurement of grain morphology and thousand kernel weight.

Fine mapping populations were grown across six environments from 2021 to 2025: E1, Ludong University, 2021–2022; E2, Muyu Village, 2021–2022; E3, Ludong University, 2023–2024; E4, Ludong University, 2024–2025; E5, Pula Valley Experimental Station 1, 2024–2025; and E6, Pula Valley Experimental Station 2, 2024–2025. KNPS was investigated by counting all grains on the main spike of each plant at the maturity stage. A randomized complete block design was used with two replications. Each plot consisted of three 2-m rows with 30 cm row spacing.

The functional annotation information of candidate genes in target regions was acquired based on the gene annotations of the Chinese Spring genome in Triticeae-Gene Tribe (http://wheat.cau.edu.cn/TGT/).

### Natural population materials, phenotypic evaluation and heritability analysis

A natural population comprising 314 wheat accessions, spanning a wide geographical range, was used for analysis of genetic effects of *qKnps-2A* and geographical distribution (Table S3). The Chinese accessions comprised landraces and modern cultivars sourced from major wheat agroecological zones across the country. The foreign accessions were selected from diverse geographical origins spanning Asia, Europe, North America, South America, Africa, and Oceania. Within the modern cultivar panel, 11, 27, 87, and 60 accessions were released during the 1980 s and earlier, 1990 s, 2000 s, and 2010 s, respectively. Field trials were conducted at five locations in northern China, namely Weifang (119.44°E, 36.68°N), Pula Valley (121.41°E, 37.31°N), Shijiazhuang (114.69°E, 37.89°N), Muyu Village (121.35°E, 37.34°N), and Ludong University (121.35°E, 37.51°N), from 2019 to 2022. All sites were characterized by natural long-day (LD) conditions. Each accession was grown in 2-m-long three-row plots with 30 cm inter-row spacing.

At maturity, six plants in the middle of each plot were selected for each genotype to investigate agronomic traits: PH, SL, peduncle length (PL), SNPS, fertile spikelet number (FSN), KNPS, TKW, GL, GW, grain compactness (GC), grain setting (GS), and yield per plant (YPP). PH was measured from the stem base to the top of the main tiller spike (excluding awns). SL was measured from the base of the spike to the tip (excluding awns). PL was measured from the base of the spike to the top node of the stem. SNPS, FSN, and KNPS were measured on the main tiller spike. GC is the ratio of KNPS to SL of the main spike. GS is the ratio of KNPS to SNPS of the main spike. TKW, GL, and GW were determined using the Intelligent Test and Analysis System (TOP Cloud-agri Technology, Zhejiang, China) from seeds after harvest. YPP was calculated as the weight of all seeds per plant.

Plot traits were determined as follows: at maturity, a 1 m^2^ area with uniform growth was selected in each plot to count the number of spikes. Twenty spikes were sampled from each plot to count grains per spike. Each plot was then harvested and threshed separately for yield calculation based on the actual harvested area. Grain quality traits were determined using a Fourier transform near-infrared (FT-NIR) spectrometer (Antaris II, Thermo Fisher Scientific, USA).

To eliminate environmental effects across multiple environments, the Best Linear Unbiased Estimator (BLUE) values for the 12 target traits in the natural population were calculated using the lme4 package in R. The phenotypic value was modeled as:

$$P_{ij}=\mu+G_i+E_j+e_{ij}$$

Where *P*_*ij*_ is the environment-mean value of the *i*-th genotype in the *j*-th environment, representing pooled replication means; *μ* is the overall mean; *G*_*i*​_ is the effect of the *i*-th genotype; *E*_*j*_​ is the random effect of the *j*-th environment; and *e*_*ij*​_ is the residual error containing both genotype-by-environment interaction and residual plots. To estimate BLUE values, genotype (*G*_*i*​_) was treated as a fixed effect. To estimate the variance components and broad-sense heritability (*H*^2^) on an entry-mean basis, the same mixed-effect model was employed, but genotype (*G*_*i*​_) was treated as a random effect. *H*^2^ was calculated as:

$${H}^{2}=\frac{{V}_{g}}{{V}_{g}+\frac{{V}_{e}}{{n}_{env}}}$$

where *V*_*g*​_ is the genotypic variance, *V*_*e*_​ is the residual variance, and *n*_*env*_​ is the number of environments.

### Transcriptome analysis

NIL-KN9204 and NIL-J411 were sampled at different stages of young spike development for transcriptome sequencing. The sampling stages were divided into early developmental stages of spike (abbreviated as EDS, including double ridge stage, glume primordium differentiation stage, floret primordium differentiation stage, and stamen and pistil primordium differentiation stage) and late developmental stages of spike (abbreviated as LDS, including anther connective formation stage, late anther connective formation stage, and tetrad stage). The classification criteria for young spike developmental stages were consistent with the quantitative grading scale described by Waddington et al. [26]. The young spike samples were collected 2–3 h after sunrise to minimize variation caused by circadian rhythms. The plants were grown in the field under natural long-day conditions. All samples were immediately frozen in liquid nitrogen and stored at − 80 °C until RNA extraction. For each biological replicate, approximately 50 young spikes at the same developmental stage were collected and pooled to reduce sampling variation. Three biological replicates were set for each sample group.

Transcriptome sequencing and analyses were performed by BGI Genomics Co., Ltd (BGI-Shenzhen, China). Total RNA extraction and strict quality assessments were performed prior to library construction. RNA quantity and integrity were assessed using a fragment analyzer (Agilent Technologies, Santa Clara, CA, USA). Library preparation was conducted with the Optimal Dual-mode mRNA Library Prep Kit (BGI-Shenzhen, China). DNBs (DNA Nanoball) were loaded onto the patterned nanoarray, and high-throughput sequencing was executed on the DNBSEQ-T7 platform utilizing a 150 bp paired-end (PE150) strategy. Raw sequencing data were filtered using SOAPnuke (version 1.5.6) to remove adapters and low-quality sequences. Transcriptome quality was evaluated using the Phred + 33 scoring system. The clean reads were aligned against the hexaploid wheat reference genome (IWGSC RefSeq v2.1) using an optimized mapping framework. To validate experimental reproducibility, unsupervised quality-control assessments were implemented. Principal Component Analysis (PCA) was performed using the vst-transformed expression matrix within the DESeq2 package. Concurrently, sample-to-sample relationships and biological replicate consistencies were evaluated using Pearson correlation coefficients (r) based on the normalized gene expression profiles. Fragments Per Kilobase of transcript per Million mapped reads (FPKM) values were calculated for transcript quantification, and DESeq2 (version 1.6.3) was employed for differentially expressed gene (DEG) analysis. Genes with a false discovery rate < 0.05 and absolute log_2_ fold change ≥ 1 were identified as DEGs.

To assess genetic background recovery and identify potential residual donor segments in the NIL pair (NIL_J411 and NIL_KN9204), we performed genome-wide SNP identification using RNA-seq datasets (pooled biological replicates from EDS and LDS stages). Raw reads were aligned to the hexaploid wheat reference genome (IWGSC RefSeq v2.1) using BWA-MEM2 (v2.3). Variant calling followed the GATK (v4.6) pipeline for RNA-seq data, including SplitNCigarReads and base quality score recalibration. High-confidence homozygous SNPs were filtered using: QUAL ≥ 30, GQ ≥ 20, and DP ≥ 5. Pairwise genetic similarity was calculated as: S = (*N*_identical_/*N*_shared_) × 100%, where *N*_identical_ is the number of identical homozygous genotypes and *N*_shared_ is the total number of shared homozygous sites. To map polymorphism distribution, SNP density was calculated using a 1 Mb sliding window. Windows with fewer than 5 SNPs were collapsed to zero to filter out sequencing background noise.

### Quantitative real-time PCR analyses

RNA from samples of NIL-KN9204 and NIL-J411 used for transcriptome sequencing was extracted using MiniBEST Plant RNA Extraction Kit (TaKaRa, Shiga, Japan). First-strand cDNA was synthesized using oligo(dT) primers and the TransScript One-Step gDNA Removal and cDNA Synthesis SuperMix (TransGen, Beijing, China).

The cDNA was diluted 5-fold for qRT-PCR. Reactions included 10 μL of 2 × TB Green Premix Ex Taq II, 0.8 μL of forward primer (10 μM), 0.8 μL of reverse primer (10 μM), and 2 μL of cDNA template in a total volume of 20 μL. Reaction conditions were 95 °C for 30 s, 40 cycles of 95 °C for 5 s and 60 °C for 34 s, followed by a melting curve with 0.2 °C steps between 60 and 95 °C. The qRT-PCR was performed using TB Green Premix Ex Taq II (TaKaRa) on an ABI PRISM 7500 real-time PCR system (Applied Biosystems, Foster City, CA, USA). Each sample was analyzed with three technical replicates, and three independent biological replicates were performed. The fluorescence threshold was set in the exponential phase and the Ct value was extracted for each sample. Expression levels of *TraesCS2A03G0902100* and *TraesCS2A03G0904700* were normalized to the housekeeping gene *glyceraldehyde-3-phosphate dehydrogenase* (*GAPDH*). Relative expression was calculated using the Pfaffl method [27], which corrects for differences in amplification efficiency between target and reference genes. Amplification efficiencies (E) were determined from standard curves and incorporated into the equation:

$$\mathrm{r}\mathrm{a}\mathrm{t}\mathrm{i}\mathrm{o}=\frac{({{E}_{target})}^{\Delta {\mathrm{C}\mathrm{T}}_{target}\left(\mathrm{c}\mathrm{o}\mathrm{n}\mathrm{t}\mathrm{r}\mathrm{o}\mathrm{l}-\mathrm{s}\mathrm{a}\mathrm{m}\mathrm{p}\mathrm{l}\mathrm{e}\right)}}{({{E}_{reference})}^{\Delta {\mathrm{C}\mathrm{T}}_{reference}\left(\mathrm{c}\mathrm{o}\mathrm{n}\mathrm{t}\mathrm{r}\mathrm{o}\mathrm{l}-\mathrm{s}\mathrm{a}\mathrm{m}\mathrm{p}\mathrm{l}\mathrm{e}\right)}}$$

### Haplotype and genetic effect analysis of candidate genes based on resequencing data

A panel of 140 wheat core germplasm accessions was utilized, comprising 127 resequencing datasets from the public database of China National Center for Bioinformation (CNCB) (https://ngdc.cncb.ac.cn/) and 13 resequencing datasets generated by our research group. All raw resequencing data were aligned to the high-quality wheat reference genome Chinese Spring 2.1 version [28] using the bwa-mem2 algorithm. Variant calling was performed following the standard pipeline of GATK4, and the final high-quality SNP and InDel genotype data were used for subsequent analysis. Haplotype analysis of the candidate genes was conducted using the R package geneHapR [29] based on variations within the coding region and the 2 kb upstream promoter region. Phenotypic evaluation for KNPS and TKW was performed on this population at the Pula Valley (121.41°E, 37.31°N) in 2024, to assess the genetic effects of different haplotypes on yield-related traits.

### Sample preparation and quantification of phytohormones

NIL-KN9204 and NIL-J411 were sampled at different stages of young spike development for phytohormone determination. The sampling stages were divided into early developmental stages of spike (abbreviated as EDS, including double ridge stage, glume primordium differentiation stage, floret primordium differentiation stage, and stamen and pistil primordium differentiation stage) and late developmental stages of spike (abbreviated as LDS, including anther connective formation stage, late anther connective formation stage, and tetrad stage). Each sample was analyzed with three technical replicates.

In this experiment, acetonitrile was used as the extraction solvent, and the QuEChERS method was employed to purify plant endogenous hormones from the samples. The hormones (IAA, ABA, Zeatin, IPR, GA1, and GA3) were then simultaneously determined by ultra-performance liquid chromatography (UPLC, Qsight LX 50, PerkinElmer, USA) coupled with triple quadrupole mass spectrometry (QQQ, Qsight 420, PerkinElmer, USA). Internal standards were added to the extraction solution for result correction. This work was done by Nanjing Ruiyuan Biotechnology Co., Ltd.

### Statistical analysis

All primers used in this study were designed with Primer Premier 5.0 software (Table S4). Sequence alignment and nucleotide variation detection were performed using DNASTAR Lasergene 7.1.0. Statistical analyses were conducted with IBM SPSS Statistics v26 (IBM Corp., Armonk, NY, USA). Comparisons between two independent groups were analyzed using Student's* t*-test. For multiple comparisons among three or more groups, Duncan's test was applied following a significant one-way ANOVA. Fisher's exact test was implemented using the fisher.test function in the R stats package to compare frequency differences of *qKnps-2A* haplotypes between modern and landrace varieties.

## Results

### Fine mapping of *qKnps-2A*

Based on the preliminary mapping results of the major QTL for kernel number per spike, *qKnps-2A*, obtained in our previous study [21] (Table S5), this study aimed to conduct fine mapping and candidate gene analysis of this locus. Whole-genome resequencing reads of the two parental lines were first independently aligned to the Chinese Spring reference genome (IWGSC RefSeq v2.1) to identify high-quality genetic variants. Within the *qKnps-2A* target interval, we detected 14,975 single-nucleotide polymorphisms (SNPs) and 1,702 insertion/deletion (InDel) polymorphisms, among which 240 exhibited length differences ≥ 5 bp (Fig. S2). Insertions/deletions ≥ 5 bp distinguishing KN9204 from J411 were identified across the *qKnps-2A* interval and its flanking sequences, yielding 25 polymorphic InDel markers (Table S1) for screening residual heterozygous lines and fine mapping of *qKnps-2A*.

To fine-map *qKnps-2A*, 428 F_7:8_ families of the KJ-RIL population were screened for residual heterozygotes with nine developed primer pairs (Table S1). Four residual heterozygous lines (RHLs)—KJ068-1, KJ150, KJ214-5 and KJ352-1—were identified (Fig. S3) and self-pollinated to generate secondary segregating populations. In the KJ352-1 progeny, plants homozygous for the KN9204 allele produced 4.89 more kernels than those carrying the J411 allele (*P* < 0.05), whereas the heterozygotes were intermediate; no significant genotypic differences were observed in the other three RHLs (*P* > 0.05) (Table S6). Consequently, the KJ352-1-derived population was selected as the secondary mapping panel for high-resolution mapping of *qKnps-2A*.

**Fig. 1:**
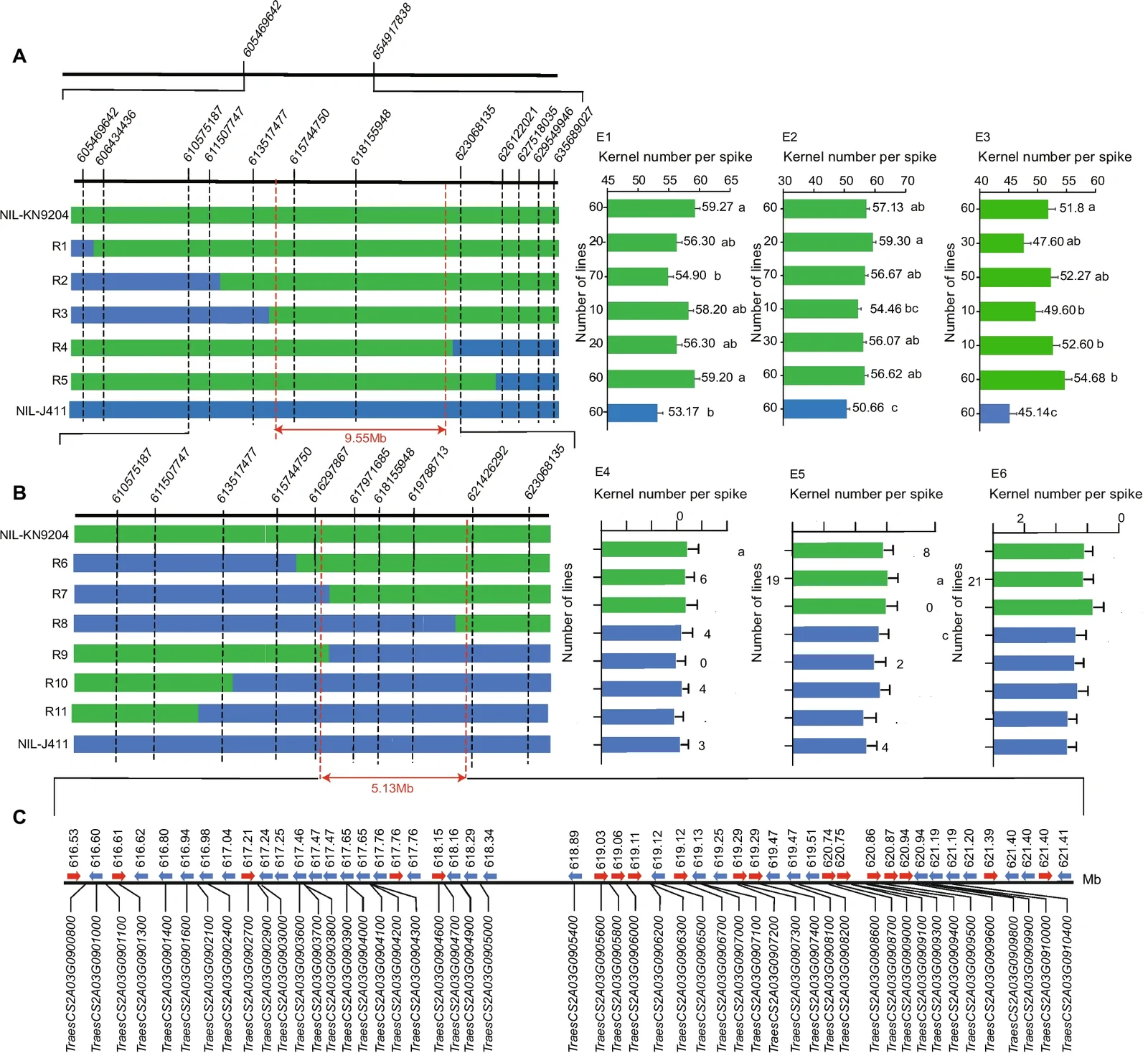
Fine mapping of *qKnps-2A*. **A**–**B** Fine mapping of *qKnps-2A* using NIL-KN9204, NIL-J411, and 11 recombinants. **C** Distribution of 50 high-confidence genes within the *qKnps-2A* mapping interval on chromosome 2 A of the Chinese Spring reference genome. Arrows indicate the transcription direction. In (**A**) and (**B**), the left panels show genotypes of the recombinants (green, NIL-KN9204; blue, NIL-J411), and the right panels show KNPS across environments. E1: Ludong University, 2021–2022; E2: Muyu Village, 2021–2022; E3: Ludong University, 2023–2024; E4: Ludong University, 2024–2025; E5: Experimental Station 1, Pula Valley, 2024–2025; E6: Experimental Station 2, Pula Valley, 2024–2025. For multiple comparisons among groups, Duncan's test was applied following a significant one-way ANOVA. Different lowercase letters indicate significant differences among groups (*P* < 0.05)

Recombinants were screened from a secondary mapping population using the aforementioned InDel markers, resulting in two near-isogenic lines (NIL-KN9204 and NIL-J411) and 11 key recombinants (R1–R11) homozygous at the target interval (Fig. 1A, B). We initially screened five recombinants (R1–R5) using twelve molecular markers from the target interval and flanking regions (Fig. 1A). Phenotypic evaluation of KNPS showed that NIL-KN9204 and R1–R5 had significantly more grains than NIL-J411 across environments E1–E3. Relative to NIL-J411, NIL-KN9204 and R1–R5 exhibited grain number increases of 6.10, 3.13, 1.73, 5.03, 3.13, and 6.03 in E1; 6.47, 8.64, 6.01, 3.80, 5.41, and 5.96 in E2; and 6.66, 2.46, 7.13, 4.46, 7.46, and 9.54 in E3, respectively. These mapping results preliminarily delimited *qKnps-2A* to a 9.55 Mb physical interval (613.52–623.07 Mb) on chromosome 2 A (Fig. 1A). Screening with densified markers within the mapped region identified six additional recombinants (R6–R11) (Fig. 1B). Further analysis revealed that R6, R7, and NIL-KN9204 had significantly higher KNPS than R8–R11 and NIL-J411 across E4–E6. Compared with NIL-KN9204, KNPS in R8–R11 and NIL-J411 was reduced by 4.71, 8.99, 4.33, 10.54, and 5.87 kernels at E4; by 2.97, 5.90, 2.16, 12.76, and 11.01 kernels at E5; and by 5.65, 6.42, 4.42, 10.59, and 10.95 kernels at E6, respectively. In contrast, R6 and R7 increased by 4.21 and 4.68 kernels at E4, 13.63 and 12.63 kernels at E5, and 10.00 and 16.31 kernels at E6 compared with NIL-J411, respectively Ultimately, *qKnps-2A* was delimited to a physical interval of 616.30–621.43 Mb (~ 5.13 Mb) on chromosome 2 A of the Chinese Spring reference genome (Fig. 1B).

According to the Chinese Spring reference genome (IWGSC RefSeq v2.1), 50 high-confidence genes were annotated within the fine-mapped interval (Fig. 1C). All except *TraesCS2A03G0904300* and *TraesCS2A03G0909900* have homologs in the KN9204 genome. The annotation of candidate genes in the target interval is provided in Table S7.

### Candidate gene analysis

To systematically prioritize the candidate genes within the *qKnps-2A* interval, we implemented a multi-dimensional filtering strategy based on the developmental biology of wheat spikes. Given that kernel number per spike is largely governed by meristem activity during spikelet and floret differentiation, we reasoned that the causal gene should exhibit differential or tissue-specific expression during early spike development. We therefore established a stepwise screening pipeline beginning with transcriptome sequencing of NIL young spikes to capture differentially expressed genes (DEGs), followed by sequential filtering through tissue-specific expression profiling, promoter and coding sequence comparison, and haplotype–trait association analysis.

**Fig. 2:**
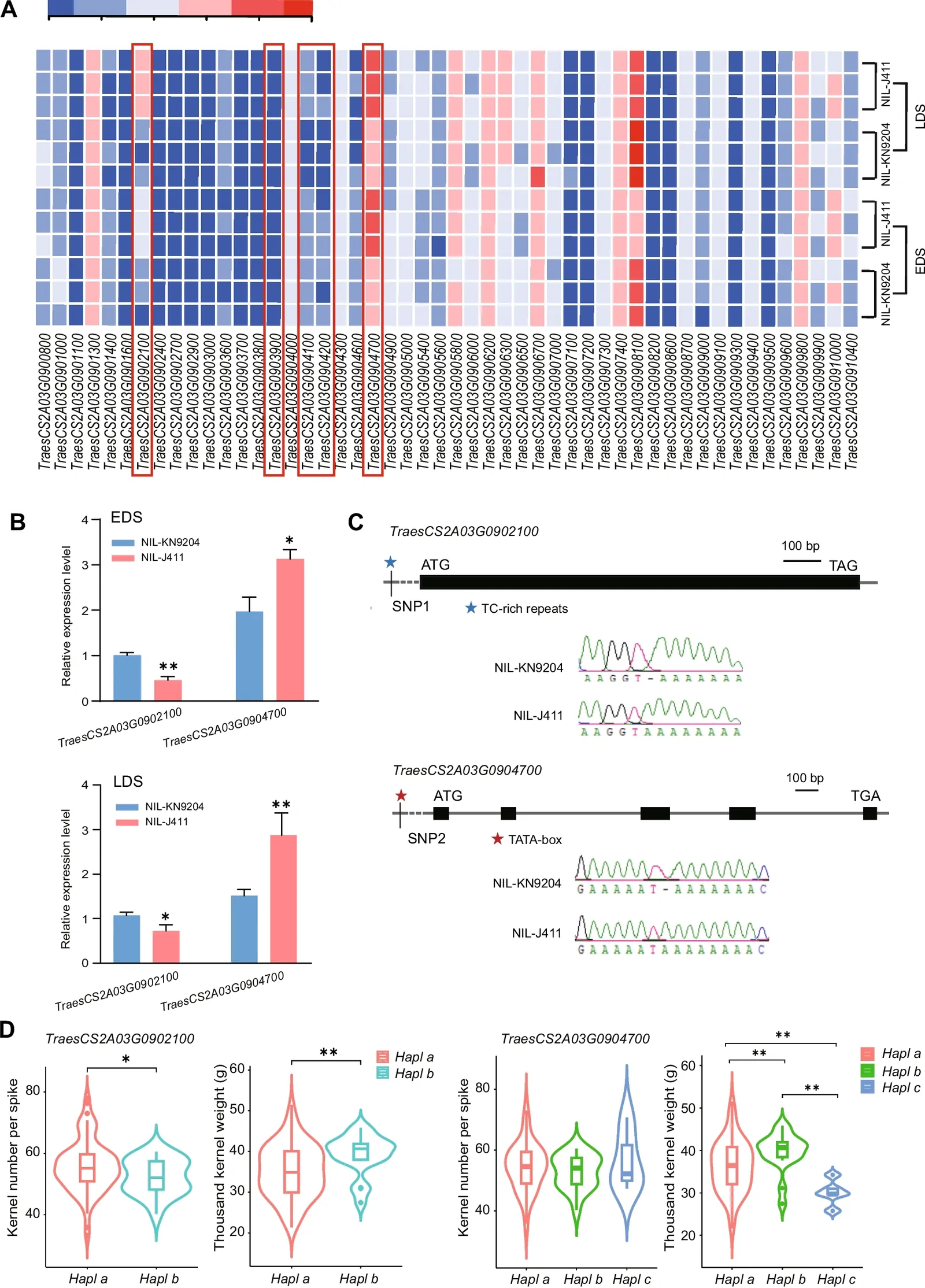
Candidate gene analysis of *qKnps-2A*. **A** Transcriptome sequencing analysis of NIL-KN9204 and NIL-J411 at different developmental stages of young spikes. Sampling stages included Early Developmental Stage (EDS) and Late Developmental Stage (LDS). The heatmap shows the expression patterns of 50 candidate genes in the *qKnps-2A* target interval. Different color intensities represent log_2_FPKM values. Differentially expressed genes are marked with red boxes. **B** Quantitative real-time PCR validation of the expression levels of differentially expressed genes *TraesCS2A03G0902100* and *TraesCS2A03G0904700*. **C** Gene structure and location of variable sites of the two candidate genes. Sequencing chromatograms display the nucleotide differences of variation sites in NIL-KN9204 and NIL-J411. Stars indicate regulatory elements affected by variation sites. **D** Haplotype analysis of the two candidate genes and effect analysis on kernel number per spike and thousand kernel weight. Significance by Student’s *t*-test: * *P* < 0.05, ** P < 0.01

As the first step of this pipeline, young spike samples from NIL-KN9204 and NIL-J411 were collected at early and late developmental stages for RNA-seq. Quality-control metrics confirmed high sequencing fidelity and strong reproducibility across biological replicates, supporting robust downstream differential expression analysis (Table S8; Fig. S4). Analysis of the early developmental stages of spike (abbreviated as EDS) revealed only two differentially expressed genes (DEGs) within the target interval: *TraesCS2A03G0902100* and *TraesCS2A03G0903900* (Fig. 2A; Table S9). At the late developmental stages of spike (abbreviated as LDS), four annotated genes showed differential expression in this region, namely *TraesCS2A03G0902100*, *TraesCS2A03G0904100*, *TraesCS2A03G0904200*, and *TraesCS2A03G0904700* (Fig. 2A; Table S10). Notably, *TraesCS2A03G0902100* was differentially expressed at both stages. qRT-PCR validation confirmed that its expression pattern was generally consistent with the RNA-seq results (Fig. 2B).

Expression profiles across tissues and developmental stages of the five candidate genes were further investigated using the Chinese Spring expression database (http://wheatomics.sdau.edu.cn/expression/index.html/) (Fig. S5). This database encompasses transcriptomic data for five tissues (root, stem, leaf, spike, and grain) at three developmental stages according to the Zadoks decimal code (root: Z10, Z13, and Z39; stem: Z30, Z32, and Z65; leaf: Z10, Z23, and Z71; spike: Z32, Z39, and Z65; grain: Z71, Z75, and Z85). Expression profiling reveals that *TraesCS2A03G0904100* and *TraesCS2A03G0904200* were highly expressed in grain but showed low expression in spikes, whereas *TraesCS2A03G0902100*, *TraesCS2A03G0903900*, and *TraesCS2A03G0904700* exhibited preferential expression in spikes. Notably, *TraesCS2A03G0903900* displayed extremely low expression across all tissues, suggesting it is an unlikely candidate. Consequently, *TraesCS2A03G0902100* and *TraesCS2A03G0904700* are considered potential candidate genes.

Based on functional annotation of candidate genes, *TraesCS2A03G0902100* encodes a zinc knuckle family protein. In *Arabidopsis*, the ZCCHC protein family is widely involved in RNA metabolism processes, including mRNA, rRNA, and microRNA [30]. In wheat, some members of this protein family are associated with spikelet number [14]. *TraesCS2A03G0904700* encodes a calcium-dependent lipid-binding domain-containing protein. *TraesCS2A03G0904700* encodes a C2 domain-containing protein. Members of this protein family may be involved in plant growth and development as well as abiotic stress responses [31].

We performed full-length amplification and sequencing of two candidate genes in NIL-KN9204 and NIL-J411. The results showed that neither candidate gene had any nucleotide variation in the coding region. For *TraesCS2A03G0902100*, a single base deletion was detected in NIL-KN9204 compared with NIL-J411 at 1596 bp upstream of the ATG start codon, located within the TC-rich repeats region (Fig. 2C). For *TraesCS2A03G0904700*, a single base deletion was identified in NIL-KN9204 relative to NIL-J411 at 948 bp upstream of the ATG, located near the TATA-box region (Fig. 2C). These promoter region variations may result in differential gene expression, thereby affecting gene function.

We used resequencing data from 140 hexaploid wheat accessions to perform haplotype analysis of the two candidate genes and to investigate the effects of different haplotypes on KNPS and TKW. The results showed that *TraesCS2A03G0902100* was divided into two haplotypes (Fig. S6A), with Hapla exhibiting increased KNPS (*P* < 0.05) and decreased TKW (*P* < 0.01) compared with Haplb (Fig. 2D). *TraesCS2A03G0904700* was classified into three haplotypes (Fig. S6B), among which no significant difference in KNPS was observed, but significant variation in TKW was detected (*P* < 0.01) (Fig. 2D). Based on these results, we infer that *TraesCS2A03G0902100* is the most likely candidate gene for *qKnps-2A*, pending further functional validation.

### Genetic analysis of* qKnps-2A* effects on yield-related and quality traits

**Fig. 3:**
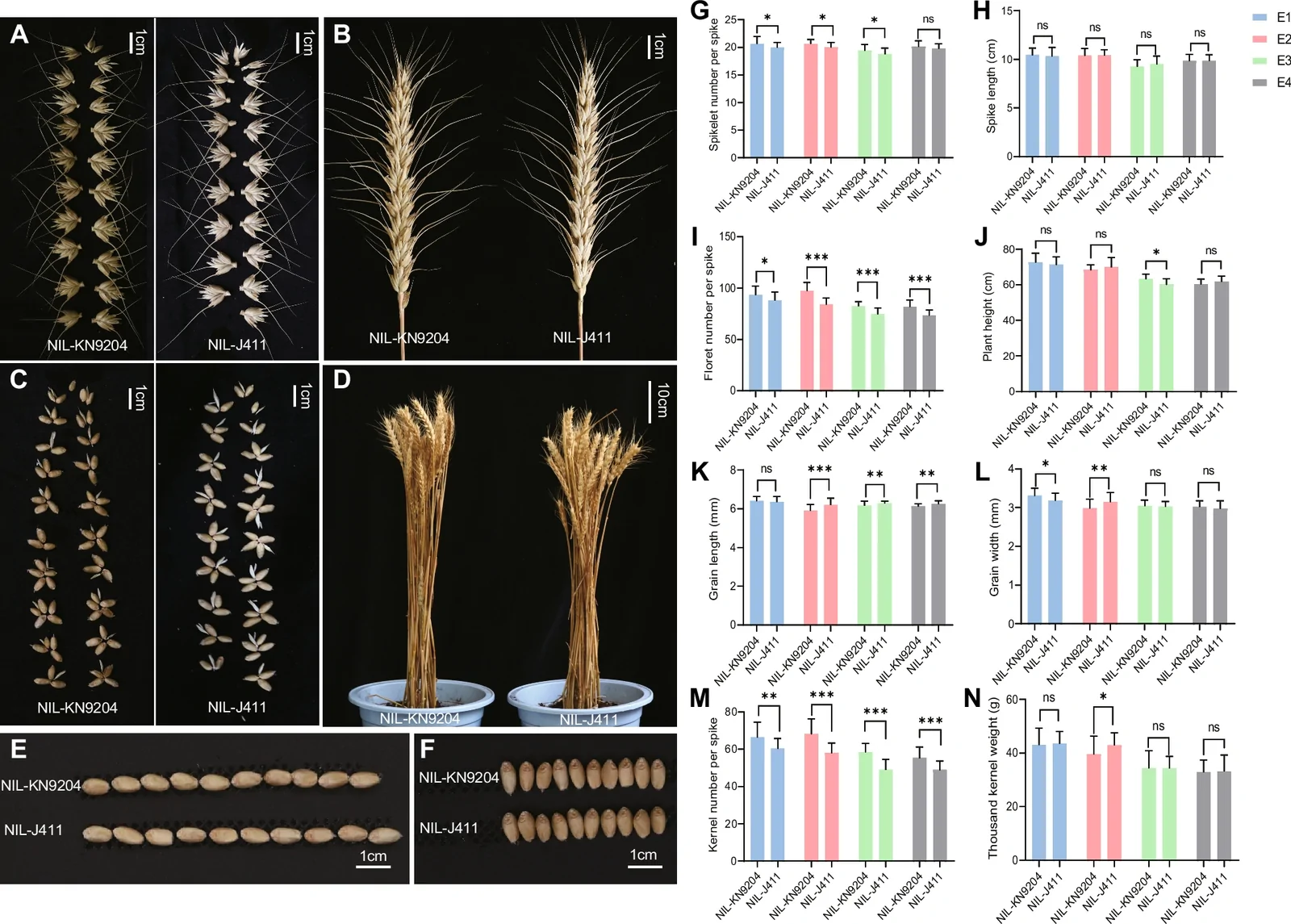
Phenotypic comparison and genetic effect analysis of *qKnps-2A* for yield-related traits between NIL-KN9204 and NIL-J411. **A** Spikelet number per spike, bar = 1 cm. **B** Spike length, bar = 1 cm. **C** Kernel number per spike, bar = 1 cm. **D** Plant height, bar = 10 cm. **E**, **F** Grain length and width, bar = 1 cm. **G**–**N** Yield-related traits across E1–E4 environments: E1, Experimental Station 1, Ludong University, Yantai, 2024–2025; E2, Experimental Station 2, Ludong University, Yantai, 2024–2025; E3, Experimental Station 1, Pula Valley, Yantai, 2024–2025; E4, Experimental Station 2, Pula Valley, Yantai, 2024–2025. Significance by Student’s* t*-test: * *P* < 0.05, ** P < 0.01, *** *P* < 0.001, n.s. *P* > 0.05

Using the target-interval near-isogenic line pair NIL-KN9204/J411, we conducted a systematic evaluation of the genetic effects of *qKnps-2A* on KNPS and other yield-related traits across multiple environments (Fig. 3A–F). The results showed that NIL-KN9204, carrying the KN9204 allele, was significantly higher than NIL-J411 for SNPS, floret number per spike (FNPS) and KNPS across all four environments, with average increases of 2.94%, 10.51% and 14.37%, respectively (Fig. 3G, I, M). Meanwhile, GL decreased by an average of 2.26% across the E2–E4 environments (Fig. 3K), whereas TKW was significantly reduced by 4.09% only in E2, with no significant differences in the other environments (Fig. 3N). No significant differences were detected between the near-isogenic lines for SL or PH (Fig. 3H, J). In summary, *qKnps-2A* primarily increases KNPS by elevating both SNPS and FNPS, accompanied by concurrent reductions in GL and TKW. These results suggest that this locus may function through pleiotropy or tight linkage with adjacent genes.

**Fig. 4:**
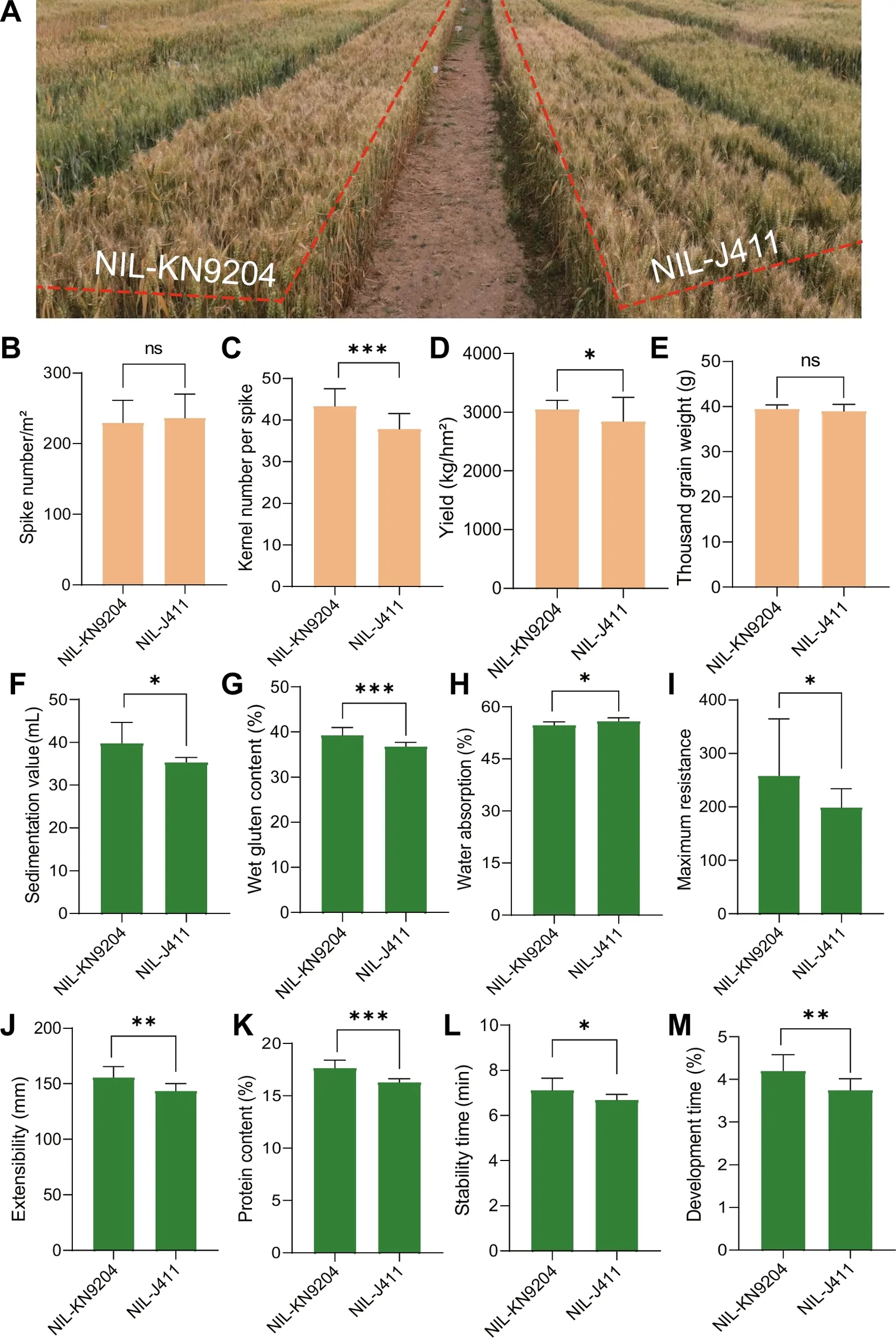
Small-plot yield and quality performance of the *qKnps-2A* near-isogenic lines. **A** Field phenotype, (**B**) Spike number per unit area, (**C**) Kernel number per spike, (**D**) Grain yield per unit area, (**E**) Thousand grain weight, (**F**) Sedimentation value, (**G**) Wet gluten content, (**H**) Water absorption, (**I**) Maximum resistance, (**J**) Extensibility, (**K**) Protein content, (**L**) Stability time, (**M**) Development time. Significance by Student’s *t*-test: * *P* < 0.05, ** P < 0.01, *** *P* < 0.001, n.s. *P* > 0.05

To further evaluate the yield potential of *qKnps-2A*, we conducted small-plot trials with three replicates using the near-isogenic line pair NIL-KN9204 and NIL-J411 (Fig. 4A). NIL-KN9204 produced significantly more kernels per spike and higher grain yield than NIL-J411, with increases of 14.56% and 7.32%, respectively (Fig. 4C, D); by contrast, neither spike number per unit area nor TKW differed significantly between the two lines (Fig. 4B, E). These results indicate that the observed yield advantage is attributable to changes in KNPS rather than to changes in grain weight. Quality trait analyses showed that NIL-KN9204 kernels exhibited significantly higher sedimentation value, wet-gluten content, maximum resistance to extension, extensibility, protein content, stability time and development time than NIL-J411, with increases of 12.62%, 6.61%, 28.57%, 8.49%, 8.33%, 6.52% and 12.18%, respectively, whereas water absorption was significantly lower (Fig. 4F–M). These results demonstrate that the superior allele derived from Kenong 9204 confers marked positive effects on both grain yield and end-use quality.

### *qKnps-2A* influences phytohormone levels during wheat spike development in NILs

**Fig. 5:**
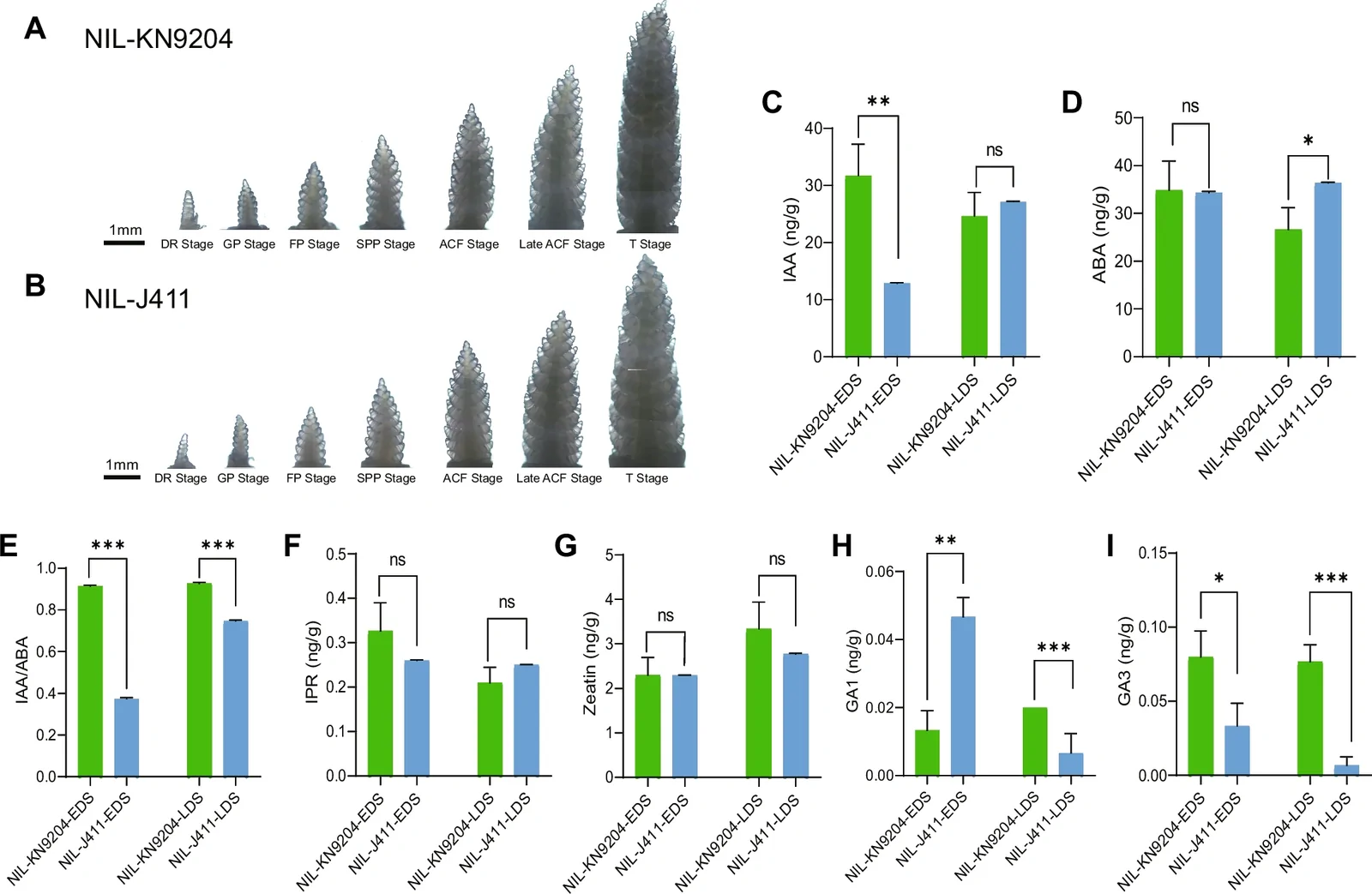
Morphology and phytohormone quantification of young spikes in *qKnps-2A* near-isogenic lines at different developmental stages. **A**–**B** Morphological features of young spikes in *qKnps-2A* NILs. DR Stage, Double Ridge Stage; GP Stage, Glume Primordium Differentiation Stage; FP Stage, Floret Primordium Differentiation Stage; SPP Stage, Stamen and Pistil Primordium Differentiation Stage; ACF Stage, Anther Connective Formation Stage; Late ACF Stage, Late Anther Connective Formation Stage; T Stage, Tetrad Stage. EDS (Early Developmental Stage) comprises DR, GP, FP and SPP stages; LDS (Late Developmental Stage) comprises ACF, late ACF and T stages. **C**–**I** Quantification of endogenous phytohormones in *qKnps-2A* NILs. IAA, ABA, IPR, zeatin, GA₁ and GA₃ were determined in both the EDS and LDS. Significance by Student’s *t*-test: * *P* < 0.05, ** P < 0.01, *** *P* < 0.001, n.s. *P* > 0.05

Spikelet differentiation and floret fertility are tightly regulated by phytohormones [12, 15, 32–34]. To investigate whether the *qKnps-2A* locus influences kernel number through hormone-mediated pathways, we quantified IAA, ABA, IPR, zeatin, GA₁ and GA₃ in the NILs during early (EDS) and late (LDS) spike development (Fig. 5A, B). During the EDS, NIL-KN9204 showed 1.46-, 1.43- and 1.52-fold higher concentrations of IAA, the IAA/ABA ratio and GA₃ (Fig. 5C, E, I), respectively, relative to NIL-J411, whereas GA₁ decreased by 72.63% (Fig. 5H) (*P* < 0.01); no significant differences were detected for ABA, IPR or zeatin (Fig. 5D, F, G). In the LDS, the IAA/ABA ratio, GA₁ and GA₃ in NIL-KN9204 rose by 0.24-, 2.26- and 14.16-fold (Fig. 5E, H, I), respectively, and ABA declined by 26.87% (Fig. 5D) (*P* < 0.01); IAA, IPR and zeatin did not differ between the two lines (Fig. 5C, F, G). Both GA₁ and GA₃ remained at trace levels (< 0.1 ng/g), suggesting that gibberellin levels differed little between the NILs and may not contribute substantially to the observed phenotypic divergence. Collectively, NIL-KN9204 and NIL-J411 exhibit distinct hormone profiles during spike development, characterized by reduced IAA availability and delayed ABA degradation in NIL-J411 compared with NIL-KN9204. These hormone differences are associated with the divergent spikelet/grain number phenotypes, though whether they are causally involved or merely correlative remains to be determined.

### Genetic effects, geographic distribution, and breeding selection of *qKnps-2A* in natural populations

To further investigate the effects of *qKnps-2A* on yield-related traits, we genotyped a natural population of 314 accessions using molecular markers tightly linked to *qKnps-2A* (Table S3), and analyzed its genetic effects by integrating multi-environment phenotypic data. Phenotypic analysis of the 314 wheat accessions across multiple environments revealed wide variations for all 12 traits (Table S11). Genotypic variance (*V*_*g*_​) was significant for all traits. Broad-sense heritability (*H*^2^) estimates across environments ranged from 0.47 (for yield per plant, YPP) to 0.97 (for plant height, PH) (Table S11). Most traits displayed high heritability (*H*^2^ > 0.75), indicating stable genetic control across different environmental conditions.

**Fig. 6:**
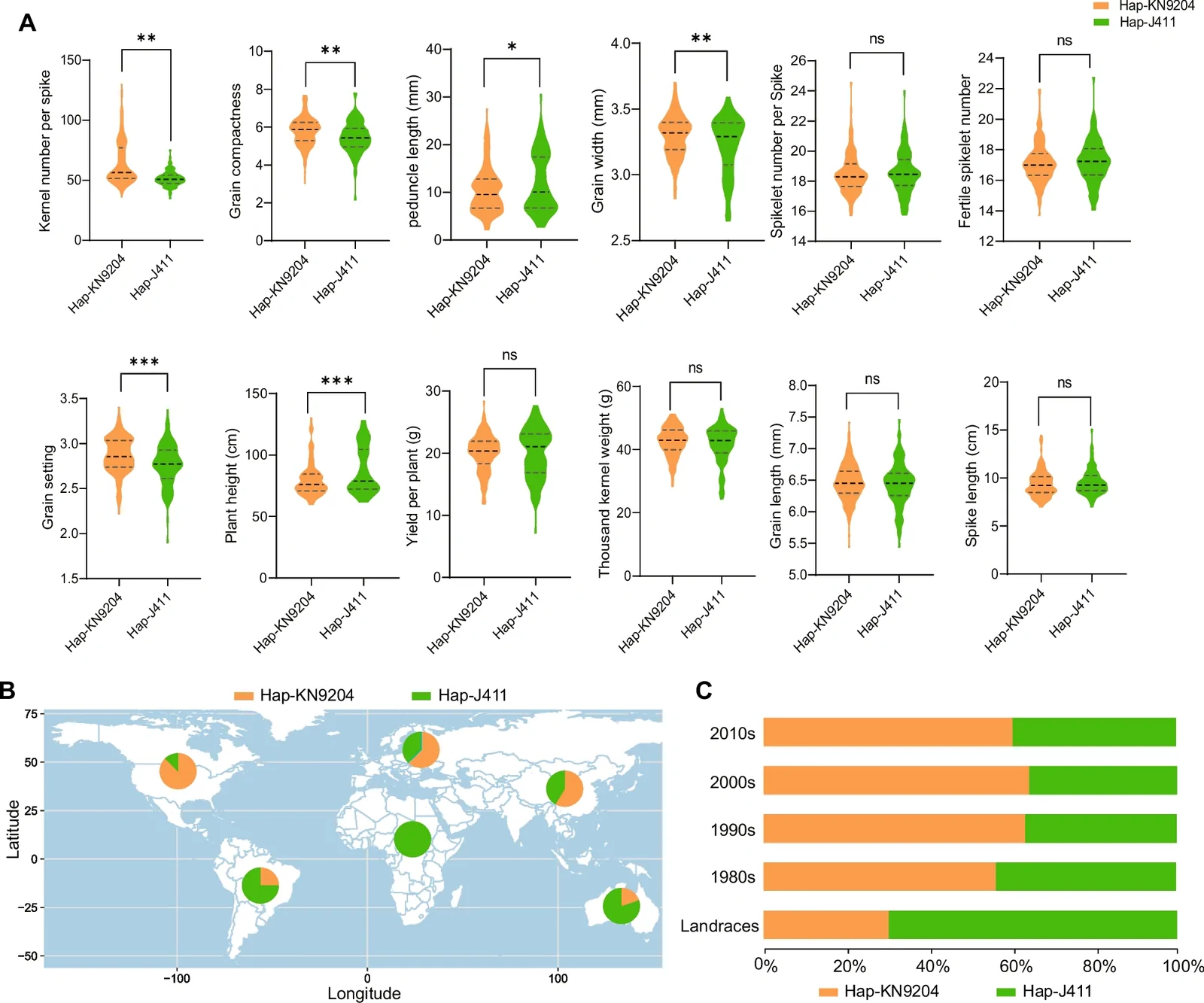
Genetic effects, geographic distribution, and breeding selection of *qKnps-2A* in natural populations. **A** Genetic effects of *qKnps-2A*. A total of 314 wheat accessions were used for phenotypic data analysis. The accessions were planted separately at five sites in northern China under natural long-day conditions during 2019–2022: Weifang, Pula Valley, Shijiazhuang, Muyu Village, and Ludong University. BLUE values were calculated using a mixed linear model with accession as a fixed effect and environment as a random effect. Data for different phenotypic traits are represented by violin plots using the BLUE values under each environment. **B** Global distribution of *qKnps-2A* haplotypes across six continents. This population comprises 314 wheat accessions from Asia, Europe, North America, South America, Africa, and Oceania. Pie charts represent the proportional distribution of different haplotypes. **C** Frequency changes of *qKnps-2A* haplotypes over decades in Chinese landraces and modern cultivars. This analysis includes 21 landraces as well as 11, 27, 87, and 60 cultivars released during the 1980 s and earlier, 1990 s, 2000 s, and 2010 s, respectively. Significance by Student’s *t*-test: * *P* < 0.05, ** P < 0.01, *** *P* < 0.001

Accessions with genotype "AA" were designated as Hap-KN9204, those with genotype "BB" as Hap-J411, and those with genotype "HH" as heterozygous type. Genotyping results showed that among the 314 accessions in the natural population, 177 carried Hap-KN9204 (56.37%), and 116 carried Hap-J411 (36.94%). Best linear unbiased estimates (BLUEs) were calculated for phenotypic data of each trait across eight environments in the natural population and used for subsequent data analysis (Fig. 6A). The results showed that Hap-KN9204 significantly increased KNPS, GC, GS, and GW compared with Hap-J411, with average increases of 3.27%, 5.67%, 3.76%, and 2.29%, respectively. However, Hap-J411 had significantly negative effects on PH and PL, with reductions of 8.86% and 12.08%, respectively. No significant differences were detected between the two haplotypes for YPP, TKW, SNPS, FSN, GL, or SL. Collectively, *qKnps-2A* influences KNPS, PH, and grain traits in wheat.

To investigate the global geographic distribution of different *qKnps-2A* haplotypes, 314 accessions from six continents were analyzed (Fig. 6B; Table S3). The results showed that Hap-KN9204 was predominant in North America, Europe, and Asia (87.50%, 62.50%, and 58.52%, respectively), whereas Hap-J411 was mainly distributed in Oceania, South America, and Africa (80.00%, 75.00%, and 100.00%, respectively). To determine whether the superior haplotype of *qKnps-2A* has been subjected to selection during wheat breeding in China, we evaluated the frequency dynamics of Hap-KN9204 and Hap-J411 among 206 wheat accessions (Fig. 6C; Table S3). The results revealed that the frequency of Hap-KN9204 increased from 30.00% in landraces to 59.62% in modern cultivars, indicating a gradual increase in its utilization. Using Fisher's Exact Test, the frequencies of Hap-KN9204 and Hap-J411 were significantly different between modern cultivars and landraces (*P* < 0.01). These results are consistent with possible positive selection of Hap-KN9204 during wheat breeding in China, although other breeding-history effects may also contribute to this pattern.

## Discussion

### Distinct genetic basis of *qKnps-2A* compared to known loci on chromosome 2A

Chromosome 2 A harbors multiple loci affecting yield-related traits, yet the causal genes within most QTL intervals remain elusive [17–22]. In this study, we fine-mapped the major stable QTL *qKnps-2A* to a 5.13 Mb interval (616.30–621.43 Mb) and identified *TraesCS2A03G0902100* as the most likely candidate gene. Comparative physical mapping indicates that this interval does not overlap with previously reported KNPS-related QTLs on chromosome 2 A [17–19, 22, 23] or with known regulators such as *WFZP* [5], *TabHLH27-A1* [35], and *TaCYP78A5-2A* [36]. Thus, *qKnps-2A* represents a genetically distinct locus.

Notably, *TraesCS2A03G0902100* encodes a Zinc knuckle (ZCCHC) family protein, a class distinct from the bHLH transcription factors or cytochrome P450 monooxygenases previously characterized on this chromosome. ZCCHC proteins are widely implicated in RNA metabolism, including mRNA stability and microRNA processing [30]. In the context of wheat spike development, preferential expression of *TraesCS2A03G0902100* in young spikes and its differential expression between NILs at both early and late developmental stages suggest that it may modulate grain number by influencing the stability or processing of transcripts involved in meristem activity or floral organogenesis. The promoter indel identified between KN9204 and J411—a single-base deletion in the TC-rich repeats region—provides a plausible regulatory polymorphism driving this expression difference. These findings expand the functional repertoire of RNA-binding proteins in wheat inflorescence development and suggest that *qKnps-2A* regulates KNPS through a pathway distinct from the hormone-mediated or transcriptional mechanisms reported for other 2 A loci.

### Stage-specific hormonal regulation of grain number by *qKnps-2A*

The determination of KNPS depends on the balance between spikelet differentiation and floret fertility, processes tightly regulated by phytohormones [12, 15, 32–34]. In this study, our physiological characterization of the NILs revealed distinct hormonal patterns correlated with the high-KNPS phenotype of NIL-KN9204. We observed that the high-KNPS allele is associated with elevated IAA levels and an increased IAA/ABA ratio during the EDS, followed by accelerated ABA degradation during the LDS. These hormone profiles are consistent with the hypothesis that *qKnps-2A* is associated with altered IAA-ABA balance—higher IAA levels during early development and lower ABA levels during late development coinciding with increased grain number. Whether these hormonal changes are causally required for or merely parallel to the increased grain number remains to be tested. The most likely candidate gene at the *qKnps-2A* locus, *TraesCS2A03G0902100*, is tentatively linked to the observed hormone differences. This gene encodes a Zinc knuckle (ZCCHC) family protein involved in RNA metabolism [30]. As a working hypothesis, altered expression might modulate hormone-related transcript stability, potentially contributing to the IAA-ABA balance differences between NILs. This proposed connection is speculative; the hormone differences could alternatively arise from other genes within the *qKnps-2A* interval or from indirect physiological feedback, and direct functional evidence is required to test any causal mechanism. Future investigation into how *qKnps-2A* interfaces with other hormone signaling modules may reveal additional layers of regulatory complexity underlying grain number determination.

### The genetic potential and breeding value of *qKnps-2A* for enhancing grain yield and quality

The KN9204 allele increased kernel number but slightly reduced grain length (2.26%) and thousand kernel weight (4.09% only in E2), indicating a potential trade-off between kernel number and kernel size. Such antagonism is well documented in wheat, where alleles at loci such as *TaGW2-A1* exert opposing effects on grain number and grain weight [11], and *TaSPL17* has been identified as a key controller of both grain number and size [37]. This aligns with the source-sink balance in cereals, where wheat grain filling is predominantly sink-limited with considerable buffering capacity against increased sink demand [38]. Nevertheless, the 7.32% yield gain suggests that the benefit of higher kernel number outweighs the minor reduction in individual grain weight under favorable conditions. The inconsistent TKW reduction across environments further suggests that this trade-off is environmentally dependent. Therefore, introgression of the KN9204 allele should be coupled with management practices or genetic backgrounds that ensure sufficient source capacity to support the increased sink demand. Despite this trade-off, the overall breeding value of *qKnps-2A* remains compelling. Unlocking the genetic potential of grain number is a primary objective in modern wheat breeding. While *TaSPL17* [37], *TaGSNE* [39], and *TaSPL13-2B* [33] have demonstrated breeding value for modulating grain number, size, or fertility, *qKnps-2A* is distinguished by its simultaneous enhancement of grain yield and end-use quality. This study systematically characterized the genetic effects, geographic distribution, and breeding selection history of the *qKnps-2A* locus. In natural populations, Hap-KN9204 significantly increased KNPS (3.27%), GC (5.67%), GS (3.76%), and GW (2.29%) compared with Hap-J411. Notably, the increase in frequency of this superior haplotype from 30.00% in landraces to 59.62% in modern cultivars is suggestive of positive selection during wheat breeding in China, although genetic drift, population bottlenecks, or hitchhiking with linked loci could also have influenced its frequency trajectory. Using target-interval near-isogenic lines, we further demonstrated that Hap-KN9204 significantly elevated SNPS (2.94%), FNPS (10.51%), and KNPS (14.37%), with small-plot trials revealing a 7.32% increase in grain yield. Importantly, this haplotype simultaneously improved multiple quality traits, including sedimentation value, wet-gluten content, and maximum resistance to extension, achieving synergistic enhancement of both yield and end-use quality. These results establish *qKnps-2A* as a valuable pleiotropic locus for wheat improvement, with Hap-KN9204 serving as an elite haplotype resource for marker-assisted selection.

Based on the findings presented here, we propose a working model for how the superior KN9204 allele at *qKnps-2A* may influence grain-number formation. The causal interval contains *TraesCS2A03G0902100*, which encodes a zinc-knuckle (ZCCHC) protein implicated in RNA metabolism. A single-base deletion in the TC-rich repeats of its promoter is associated with differential expression of this gene during both early and late spike development in NILs. These expression differences coincide with altered phytohormone profiles that are associated with increased spikelet number, floret number, and ultimately kernel number per spike. The Hap-KN9204 haplotype carrying this promoter variant is prevalent in modern cultivars and correlates with higher KNPS and improved grain quality in global germplasm. We therefore hypothesize that the promoter polymorphism modulates *TraesCS2A03G0902100* expression, which may influence RNA metabolic processes associated with hormone homeostasis during spike development, thereby contributing to the increased grain-number phenotype. Further experiments, such as transgenic complementation, CRISPR-based gene editing, and transcript stability assays, will be required to test this proposed mechanism.

## Supplementary Information

Supplementary Material 1: Fig. S1 Genome-wide SNP density distribution between the NIL pair at two developmental stages. (A) Physical distribution of polymorphic SNPs between NIL_J411 and NIL_KN9204 at the early developing spike (EDS) stage. (B) Physical distribution of polymorphic SNPs between NIL_J411 and NIL_KN9204 at the late developing spike (LDS) stage.The x-axis represents physical distance (Mb) along the 21 wheat chromosomes based on the Chinese Spring genome (v2.1). The y-axis lists the chromosomes from top to bottom. The color scale represents the number of polymorphic SNPs within a 1 Mb sliding window, with a minimum threshold of 5 SNPs to suppress background sequencing noise. Gray bars indicate genomic regions identical to the recurrent background.

Supplementary Material 2: Fig. S2 Distribution of InDel (≥5 bp) polymorphic sites within the *qKnps-2A* target interval.

Supplementary Material 3: Fig. S3 Identification of residual heterozygous lines within the* qKnps-2A* target interval. Yellow segments denote heterozygous regions, green segments the J411 genotype, and red segments the KN9204 genotype; the left scale indicates Chinese Spring physical positions (IWGSC RefSeq v2.1), with the positions of InDel5 and InDel7 marked by red boxes.

Supplementary Material 4: Fig. S4 Unsupervised quality-control diagnostics of the RNA-seq datasets across 12 wheat samples. (A) Principal Component Analysis (PCA) score plot based on the vst-transformed normalized counts. The horizontal and vertical axes indicate the percentage of variance explained by principal component 1 (PC1: 46%) and principal component 2 (PC2: 35%), respectively. Samples are color-coded by experimental groups, and biological replicates group tightly together. (B) Global sample-to-sample correlation matrix based on Pearson correlation coefficients (*r*). The color scale reflects the correlation strength, where dark red denotes perfect positive correlation (*r*= 1.000) and dark blue signifies lower relative correlation. Dendrograms on the top and left represent hierarchical clustering based on Euclidean distance.

Supplementary Material 5: Fig. S5 Expression pattern analysis of *qKnps-2A* candidate genes across tissues and developmental stages based on the Chinese Spring gene expression database. This database encompasses transcriptomic data for five tissues (root, stem, leaf, spike, and grain) at three developmental stages according to the Zadoks decimal code: (A) *TraesCS2A03G0902100*; (B) *TraesCS2A03G0903900*; (C) *TraesCS2A03G0904100*; (D)*TraesCS2A03G0904200*; (E) *TraesCS2A03G0904700*.

Supplementary Material 6: Fig. S6 Polymorphic sites and haplotype analysis of two candidate genes based on resequencing data from 140 accessions. (A) *TraesCS2A03G0902100*; (B) *TraesCS2A03G0904700*.

Supplementary Material 7: Table S1-S11.

## Data Availability

All data supporting the results of this study are available within the paper and its supplementary data published online. The plant materials used in this study are available from the corresponding author upon reasonable request. The transcriptome datasets generated during the current study are available in the NCBI Sequence Read Archive (SRA) repository under BioProject accession number PRJNA1452205 (https://www.ncbi.nlm.nih.gov/sra/PRJNA1452205).

## References

[1] FAO. Food outlook: biannual report on global food markets (statistical appendix, wheat statistics). Rome: FAO; 2022.

[2] Xiao J, Liu B, Yao Y, et al. Wheat genomic study for genetic improvement of traits in China. Sci China Life Sci. 2022;65:1718–75. 10.1007/s11427-022-2178-7. 36018491

[3] Quarrie S, Pekic Quarrie S, Radosevic R, et al. Dissecting a wheat QTL for yield present in a range of environments: from the QTL to candidate genes. J Exp Bot. 2006;57:2627–37. 10.1093/jxb/erl026. 16831847

[4] Li Y, Li L, Zhao M, et al. Wheat FRIZZY PANICLE activates VERNALIZATION1-A and HOMEOBOX4-A to regulate spike development in wheat. Plant Biotechnol J. 2021;19:1141–54. 10.1111/pbi.13535. PMC8196646 33368973

[5] Du D, Zhang D, Yuan J, et al. FRIZZY PANICLE defines a regulatory hub for simultaneously controlling spikelet formation and awn elongation in bread wheat. New Phytol. 2021;231:814–33. 10.1111/nph.17388. 33837555

[6] Boden SA, Cavanagh C, Cullis BR, et al. Ppd-1 is a key regulator of inflorescence architecture and paired spikelet development in wheat. Nat Plants. 2015;1:14016. 10.1038/nplants.2014.16. 27246757

[7] Dixon LE, Greenwood JR, Bencivenga S, et al. TEOSINTE BRANCHED1 regulates inflorescence architecture and development in bread wheat (*Triticum aestivum*). Plant Cell. 2018;30:563–81. 10.1105/tpc.17.00961. PMC5894836 29444813

[8] Chen Z, Ke W, He F, et al. A single nucleotide deletion in the third exon of FT-D1 increases the spikelet number and delays heading date in wheat (*Triticum aestivum* L.). Plant Biotechnol J. 2022;20:920–33. 10.1111/pbi.13773. PMC9055817 34978137

[9] Zhang X, Jia H, Li T, et al. TaCol-B5 modifies spike architecture and enhances grain yield in wheat. Science. 2022;376:180–3. 10.1126/science.abm0717. 35389775

[10] Sakuma S, Golan G, Guo Z, et al. Unleashing floret fertility in wheat through the mutation of a homeobox gene. Proc Natl Acad Sci U S A. 2019;116:5182–7. 10.1073/pnas.1815465116. PMC6421441 30792353

[11] Zhai H, Feng Z, Du X, et al. A novel allele of TaGW2-A1 is located in a finely mapped QTL that increases grain weight but decreases grain number in wheat (*Triticum aestivum* L.). Theor Appl Genet. 2018;131:539–53. 10.1007/s00122-017-3017-y. PMC5814529 29150697

[12] Huang X, Qian Q, Liu Z, et al. Natural variation at the DEP1 locus enhances grain yield in rice. Nat Genet. 2009;41:494–7. 10.1038/ng.352. 19305410

[13] Kong X, Wang F, Geng S, et al. The wheat AGL6-like MADS-box gene is a master regulator for floral organ identity and a target for spikelet meristem development manipulation. Plant Biotechnol J. 2022;20:75–88. 10.1111/pbi.13696. PMC8710900 34487615

[14] Wang Y, Yu H, Tian C, et al. Transcriptome association identifies regulators of wheat spike architecture. Plant Physiol. 2017;175:746–57. 10.1104/pp.17.00694. PMC5619896 28807930

[15] Zhang J, Liu W, Yang X, et al. Isolation and characterization of two putative cytokinin oxidase genes related to grain number per spike phenotype in wheat. Mol Biol Rep. 2011;38:2337–47. 10.1007/s11033-010-0367-9. 21104150

[16] Yao FQ, Li XH, Wang H, et al. Down-expression of TaPIN1s increases the tiller number and grain yield in wheat. BMC Plant Biol. 2021;21:443. 10.1186/s12870-021-03217-w. PMC8482684 34592922

[17] Li S, Jia J, Wei X, et al. A intervarietal genetic map and QTL analysis for yield traits in wheat. Mol Breed. 2007;20:167–78. 10.1007/s11032-007-9080-3.

[18] Cui F, Zhao C, Ding A, et al. Construction of an integrative linkage map and QTL mapping of grain yield-related traits using three related wheat RIL populations. Theor Appl Genet. 2014;127:659–75. 10.1007/s00122-013-2249-8. 24326459

[19] Bhusal N, Sarial AK, Sharma P, et al. Mapping QTLs for grain yield components in wheat under heat stress. PLoS ONE. 2017;12:e189594. 10.1371/journal.pone.0189594. PMC5736223 29261718

[20] Shi W, Hao C, Zhang Y, et al. A combined association mapping and linkage analysis of kernel number per spike in common wheat (*Triticum aestivum* L). Front Plant Sci. 2017;8:1412. 10.3389/fpls.2017.01412. PMC5563363 28868056

[21] Qin R, Ma T, Cai Y, et al. Characterization and fine mapping analysis of a major stable QTL qKnps-4A for kernel number per spike in wheat. Theor Appl Genet. 2023;136(10):211. 10.1007/s00122-023-04456-1. 37737910

[22] Lee HS, Jung J, Kang C, et al. Mapping of QTL for yield and its related traits in a doubled haploid population of Korean wheat. Plant Biotechnol Rep. 2014;8:443–54. 10.1007/s11816-014-0337-0.

[23] Cao S, Xu D, Hanif M, et al. Genetic architecture underpinning yield component traits in wheat. Theor Appl Genet. 2020;133:1811–23. 10.1007/s00122-020-03562-8. 32062676

[24] Shi X, Cui F, Han X, et al. Comparative genomic and transcriptomic analyses uncover the molecular basis of high nitrogen-use efficiency in the wheat cultivar Kenong 9204. Mol Plant. 2022;15:1440–56. 10.1016/j.molp.2022.07.008. 35864747

[25] Ma S, Wang M, Wu J, et al. WheatOmics: a platform combining multiple omics data to accelerate functional genomics studies in wheat. Mol Plant. 2021;14:1965–8. 10.1016/j.molp.2021.10.006. 34715393

[26] Waddington SR, Cartwright PM, Wall PC. A quantitative scale of spike initial and pistil development in barley and wheat. Ann Bot. 1983;51(1):119–30. 10.1093/oxfordjournals.aob.a086434.

[27] Pfaffl MW. A new mathematical model for relative quantification in real-time RT-PCR. Nucleic Acids Res. 2001;29(9):e45. 10.1093/nar/29.9.e45. PMC55695 11328886

[28] Zhu T, Wang L, Rimbert H, et al. Optical maps refine the bread wheat *Triticum aestivum* cv. Chinese Spring genome assembly. Plant J. 2021;107:303–14. 10.1111/tpj.15289. PMC8360199 33893684

[29] Zhang R, Jia G, Diao X. GeneHapR: an R package for gene haplotypic statistics and visualization. BMC Bioinformatics. 2023;24(1):199. 10.1186/s12859-023-05318-9. PMC10186671 37189023

[30] Aceituno-Valenzuela U, Micol-Ponce R, Ponce MR. Genome-wide analysis of CCHC-type zinc finger (ZCCHC) proteins in yeast, *Arabidopsis*, and humans. Cell Mol Life Sci. 2020;77(20):3991–4014. 10.1007/s00018-020-03518-7. PMC11105112 32303790

[31] Qin T, Tian Q, Wang G, et al. Lower temperature 1 enhances ABA responses and plant drought tolerance by modulating the stability and localization of C2-domain ABA-related proteins in *Arabidopsis*. Mol Plant. 2019;12(9):1243–58. 10.1016/j.molp.2019.05.002. 31102784

[32] Liu Y, Zhang L, Zhu A, et al. Multi-omics identifies key genetic and metabolic networks regulating spike organ development in wheat. Plant Cell. 2025;37(11):koaf250. 10.1093/plcell/koaf250. 41108752

[33] Li L, Shi F, Wang Y, et al. Wheat TaSPL13-2B improves floret fertility and enhances grain number per spikelet through jasmonic acid signalling pathway. Plant Biotechnol J. 2025;0:1–20. 10.1111/pbi.70463. PMC13140700 41257535

[34] Guo X, Zhang Z, Li J, et al. Phenotypic and transcriptome profiling of spikes reveals the regulation of light regimens on spike growth and fertile floret number in wheat. Plant Cell Environ. 2024;47(5):1575–91. 10.1111/pce.14832. 38269615

[35] Wang D, Zhang X, Cao Y, et al. TabHLH27 orchestrates root growth and drought tolerance to enhance water use efficiency in wheat. J Integr Plant Biol. 2024;66(7):1295–312. 10.1111/jipb.13670. 38695649

[36] Guo L, Ma M, Wu L, et al. Modified expression of TaCYP78A5 enhances grain weight with yield potential by accumulating auxin in wheat (*Triticum aestivum* L.). Plant Biotechnol J. 2022;20(1):168–82. 10.1111/pbi.13704. PMC8710830 34510688

[37] Liu Y, Chen J, Yin C, et al. A high-resolution genotype-phenotype map identifies the TaSPL17 controlling grain number and size in wheat. Genome Biol. 2023;24(1):196. 10.1186/s13059-023-03044-2. PMC10463835 37641093

[38] Borrás L, Slafer GA, Otegui ME. Seed dry weight response to source–sink manipulations in wheat, maize and soybean: a quantitative reappraisal. Field Crops Res. 2004;86:131–46. 10.1016/j.fcr.2003.08.002.

[39] Khan N, Zhang Y, Wang J, et al. TaGSNE, a WRKY transcription factor, overcomes the trade-off between grain size and grain number in common wheat and is associated with root development. J Exp Bot. 2022;73(19):6678–96. 10.1093/jxb/erac327. 35906966

