# Supplementary material for: Fine mapping and candidate gene analysis of qKnps-2A for kernel number per spike in wheat
Source: BMC Plant Biol. 2026 Aug 11;26:1911. doi: 10.1186/s12870-026-09727-9 (PMC13746720; doi:10.1186/s12870-026-09727-9)
Supplement: Supplementary file 1 [file 12870_2026_9727_MOESM1_ESM.pdf]

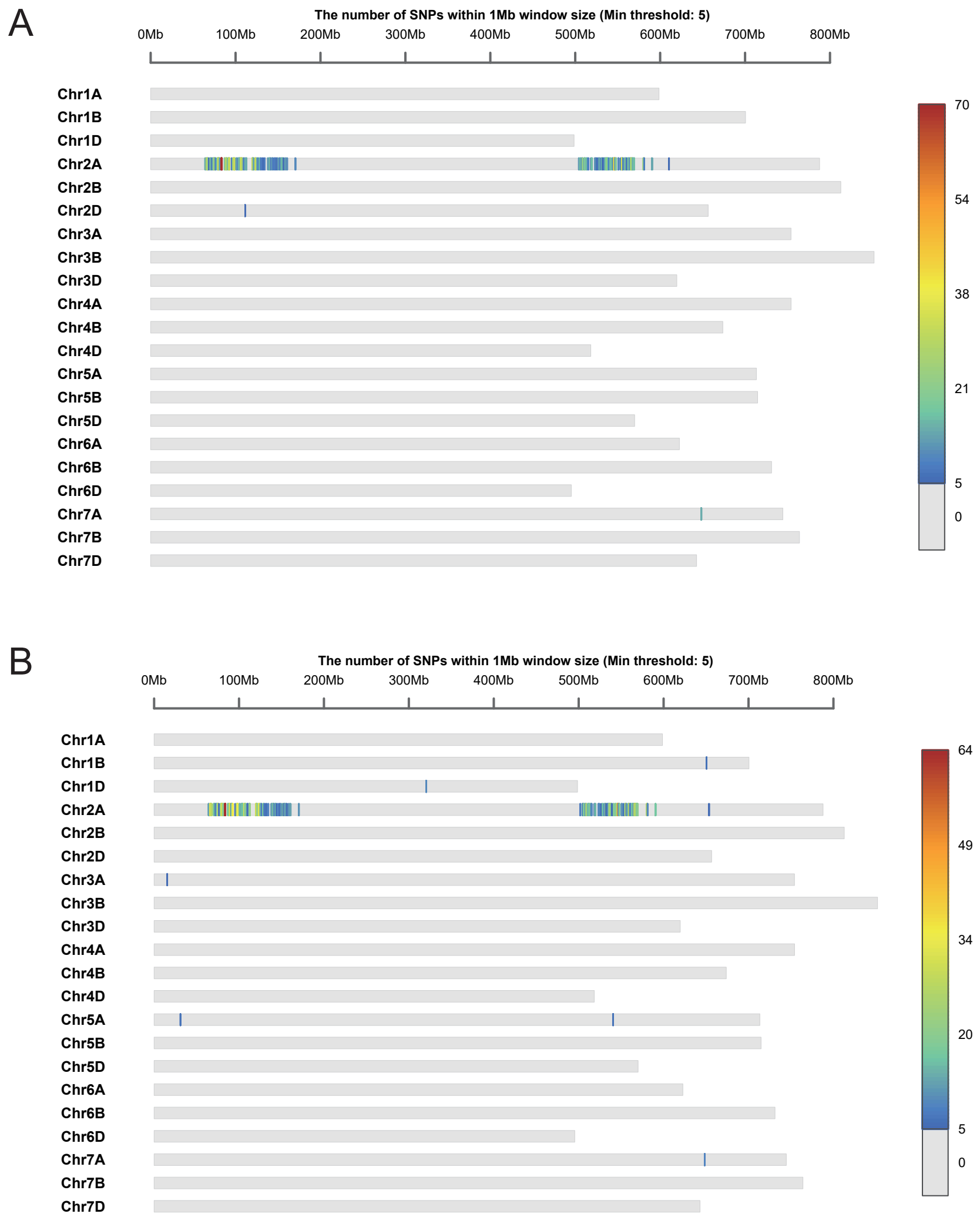

Fig. S1 Genome-wide SNP density distribution between the NIL pair at two developmental stages. **(A)** Physical distribution of polymorphic SNPs between NIL_J411 and NIL_KN9204 at the early developing spike (EDS) stage. **(B)** Physical distribution of polymorphic SNPs between NIL_J411 and NIL_KN9204 at the late developing spike (LDS) stage. The x-axis represents physical distance (Mb) along the 21 wheat chromosomes based on the Chinese Spring genome (v2.1). The y-axis lists the chromosomes from top to bottom. The color scale represents the number of polymorphic SNPs within a 1 Mb sliding window, with a minimum threshold of 5 SNPs to suppress background sequencing noise. Gray bars indicate genomic regions identical to the recurrent background.
