# Supplementary material for: Fine mapping and candidate gene analysis of qKnps-2A for kernel number per spike in wheat
Source: BMC Plant Biol. 2026 Aug 11;26:1911. doi: 10.1186/s12870-026-09727-9 (PMC13746720; doi:10.1186/s12870-026-09727-9)
Supplement: Supplementary file 2 [file 12870_2026_9727_MOESM2_ESM.pdf]

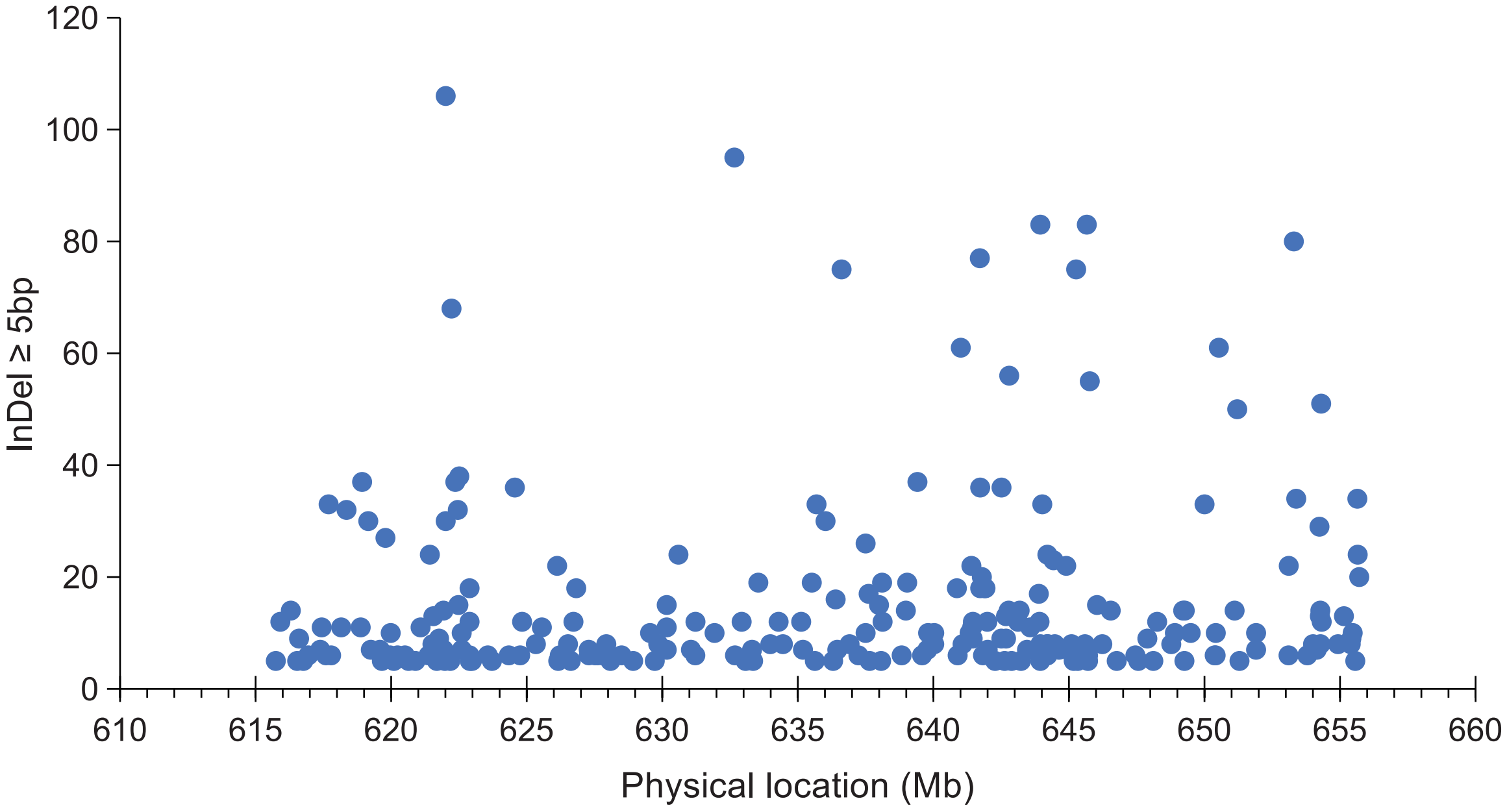

Fig. S2 Distribution of InDel (≥5 bp) polymorphic sites within the *qKnps-2A* target interval.
