# Supplementary material for: Fine mapping and candidate gene analysis of qKnps-2A for kernel number per spike in wheat
Source: BMC Plant Biol. 2026 Aug 11;26:1911. doi: 10.1186/s12870-026-09727-9 (PMC13746720; doi:10.1186/s12870-026-09727-9)
Supplement: Supplementary file 3 [file 12870_2026_9727_MOESM3_ESM.pdf]

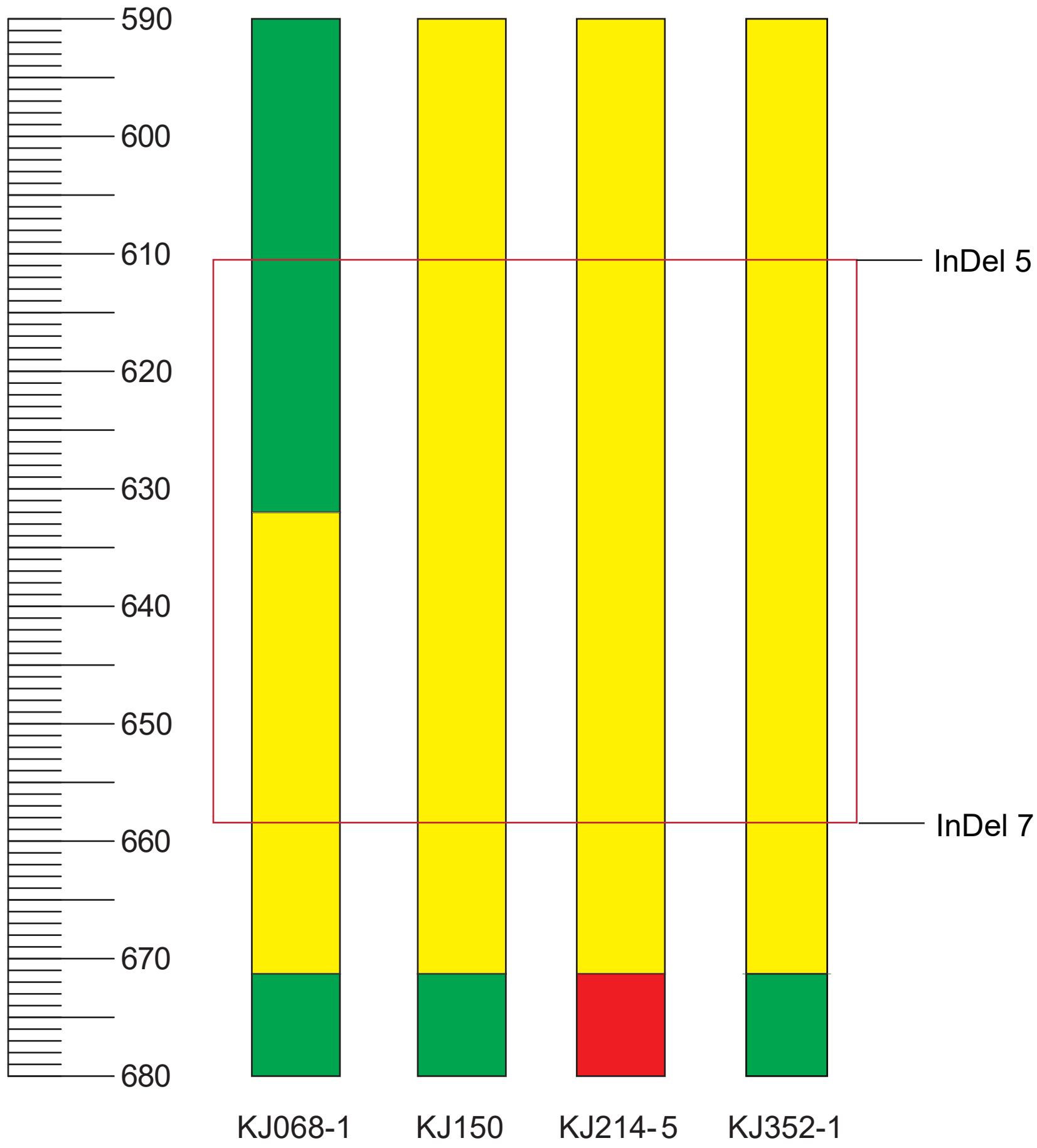

Fig. S3 Identification of residual heterozygous lines within the *qKnps-2A* target interval. Yellow segments denote heterozygous regions, green segments the J411 genotype, and red segments the KN9204 genotype; the left scale indicates Chinese Spring physical positions (IWGSC RefSeq v2.1), with the positions of InDel5 and InDel7 marked by red boxes.
