# Supplementary material for: Fine mapping and candidate gene analysis of qKnps-2A for kernel number per spike in wheat
Source: BMC Plant Biol. 2026 Aug 11;26:1911. doi: 10.1186/s12870-026-09727-9 (PMC13746720; doi:10.1186/s12870-026-09727-9)
Supplement: Supplementary file 4 [file 12870_2026_9727_MOESM4_ESM.pdf]

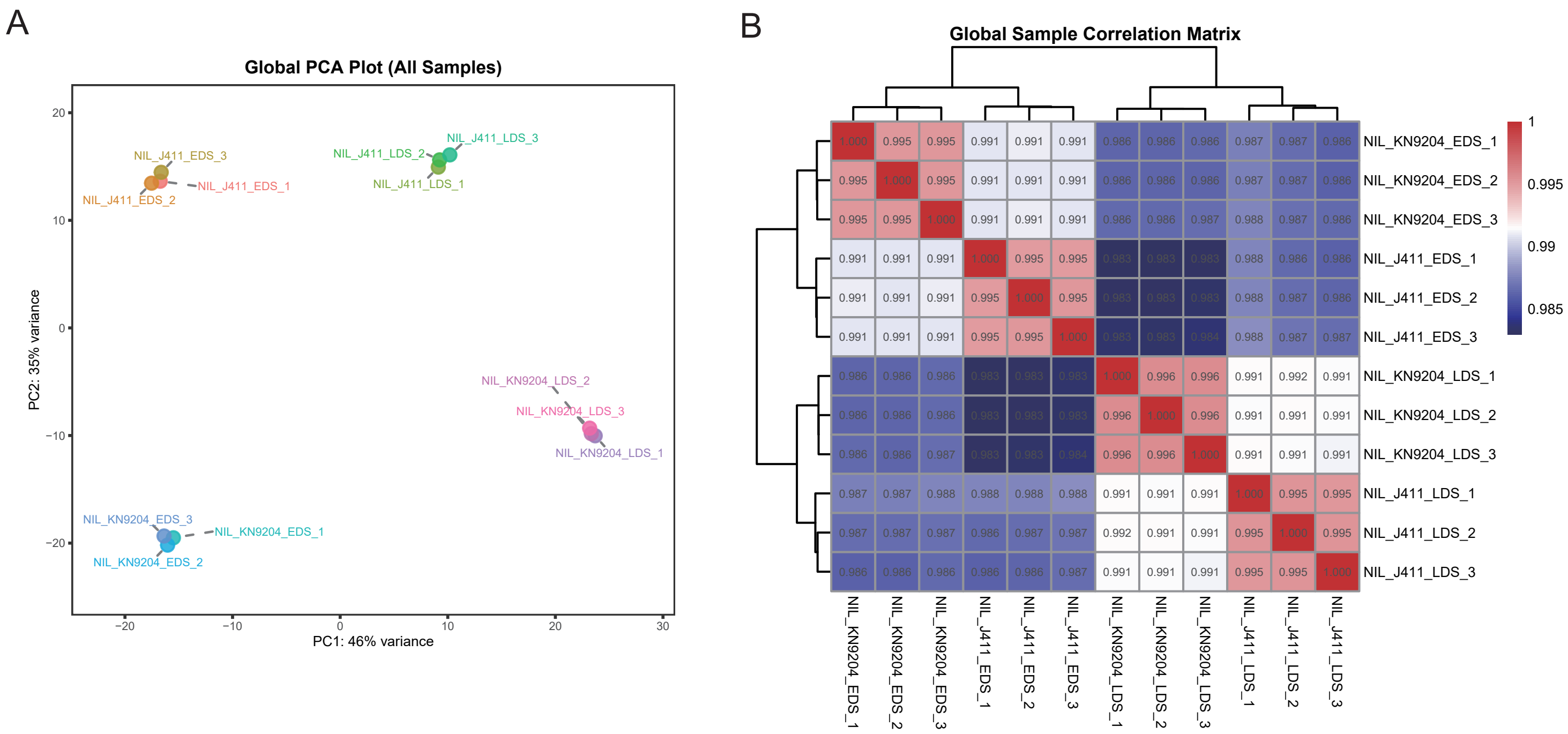

Fig. S4 Unsupervised quality-control diagnostics of the RNA-seq datasets across 12 wheat samples. (A) Principal Component Analysis (PCA) score plot based on the vst-transformed normalized counts. The horizontal and vertical axes indicate the percentage of variance explained by principal component 1 (PC1: 46%) and principal component 2 (PC2: 35%), respectively. Samples are color-coded by experimental groups, and biological replicates group tightly together. **(B)** Global sample-to-sample correlation matrix based on Pearson correlation coefficients (*r*). The color scale reflects the correlation strength, where dark red denotes perfect positive correlation (*r* = 1.000) and dark blue signifies lower relative correlation. Dendrograms on the top and left represent hierarchical clustering based on Euclidean distance.
