# Supplementary material for: Fine mapping and candidate gene analysis of qKnps-2A for kernel number per spike in wheat
Source: BMC Plant Biol. 2026 Aug 11;26:1911. doi: 10.1186/s12870-026-09727-9 (PMC13746720; doi:10.1186/s12870-026-09727-9)
Supplement: Supplementary file 5 [file 12870_2026_9727_MOESM5_ESM.pdf]

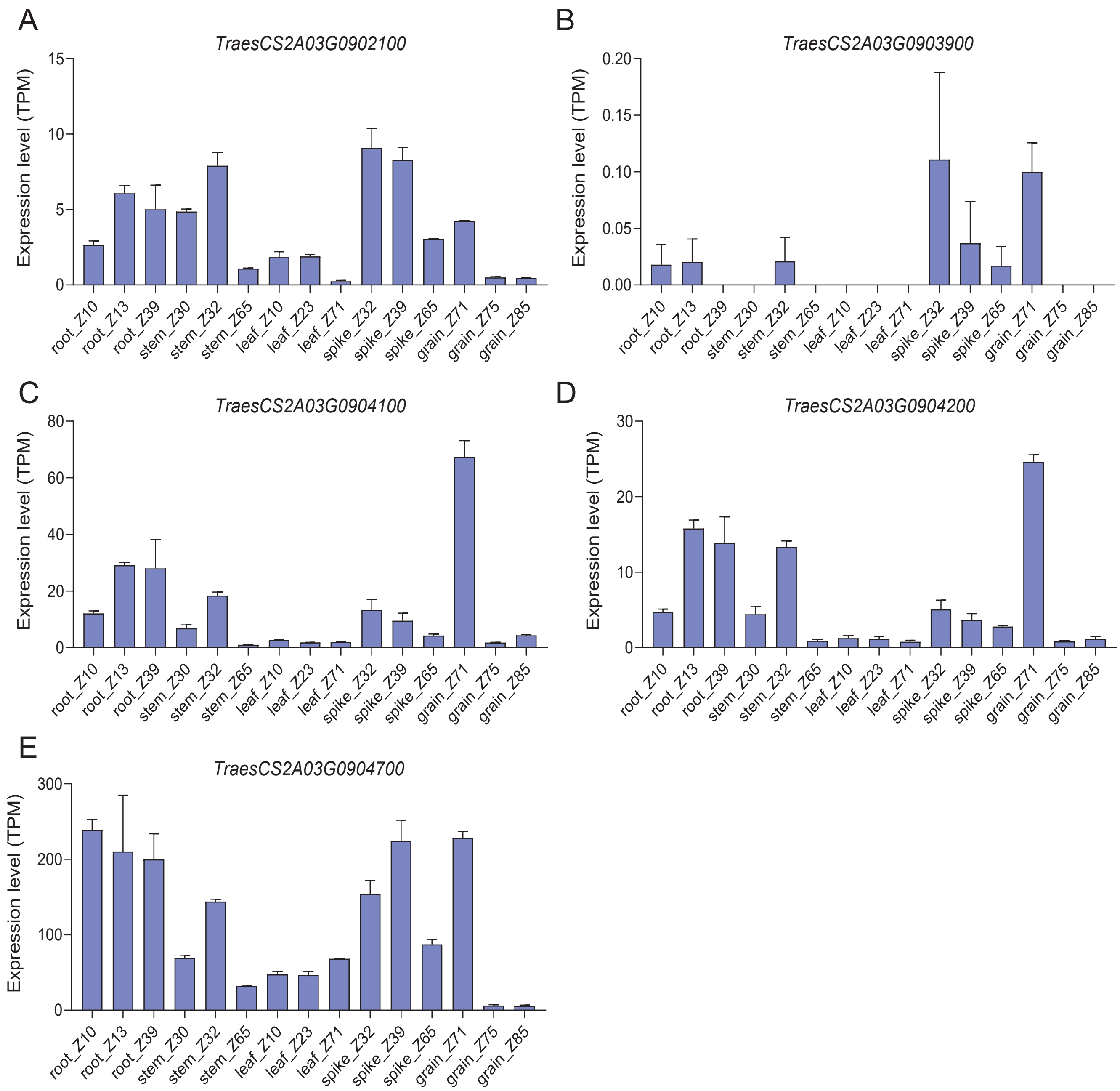

Fig. S5 Spatiotemporal expression pattern analysis of *qKnps-2A* candidate genes based on the Chinese Spring gene expression database. This database encompasses transcriptomic data for five tissues (root, stem, leaf, spike, and grain) at three developmental stages according to the Zadoks decimal code: **(A)** *TraesCS2A03G0902100*; **(B)** *TraesCS2A03G0903900*; **(C)** *TraesCS2A03G0904100*; **(D)** *TraesCS2A03G0904200*; **(E)** *TraesCS2A03G0904700*.
