# Supplementary material for: Fine mapping and candidate gene analysis of qKnps-2A for kernel number per spike in wheat
Source: BMC Plant Biol. 2026 Aug 11;26:1911. doi: 10.1186/s12870-026-09727-9 (PMC13746720; doi:10.1186/s12870-026-09727-9)
Supplement: Supplementary file 6 [file 12870_2026_9727_MOESM6_ESM.pdf]

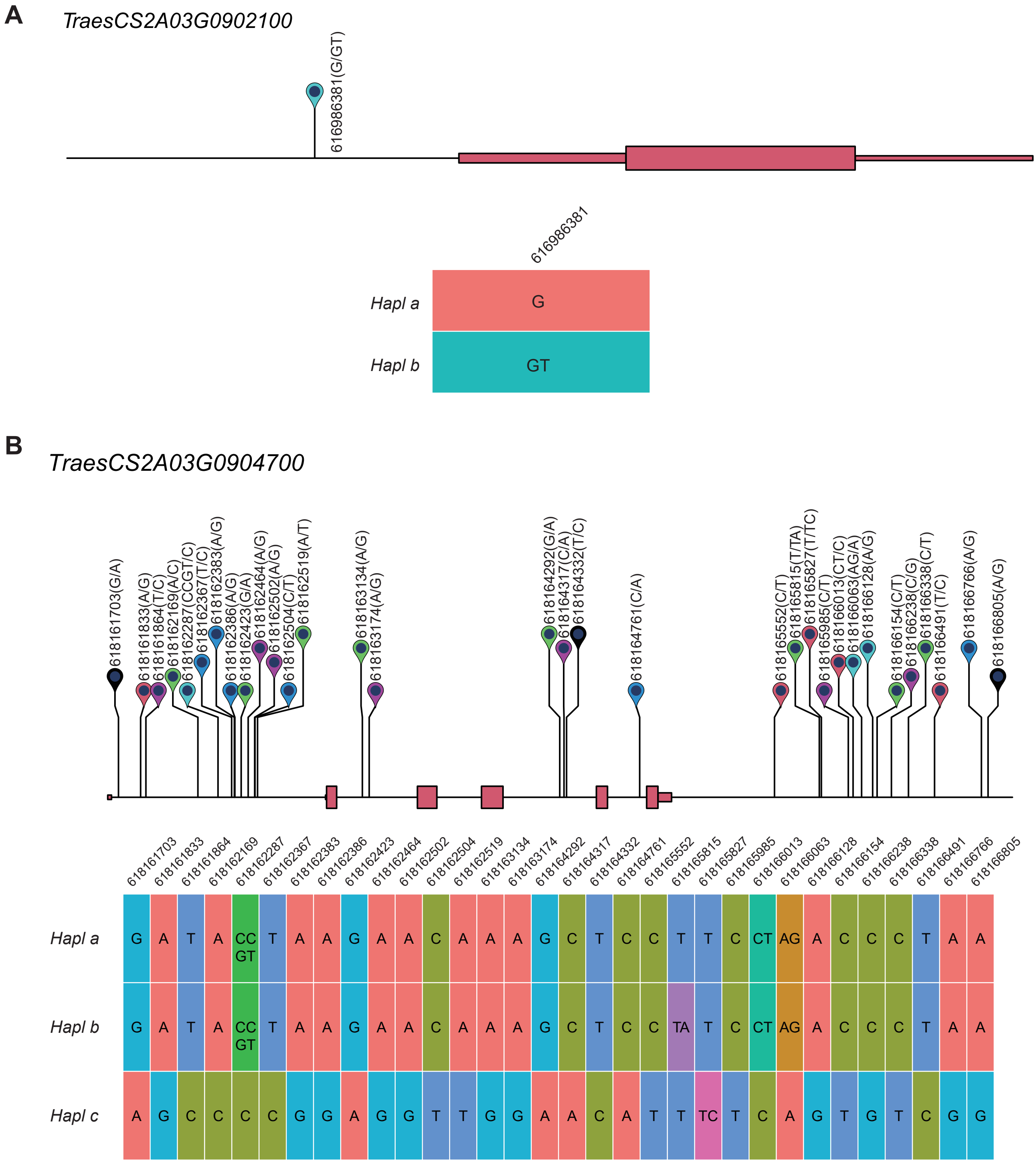

Fig. S6 Polymorphic sites and haplotype analysis of two candidate genes based on resequencing data from 140 accessions. **(A)** *TraesCS2A03G0902100*; **(B)** *TraesCS2A03G0904700*.
